# Hierarchies of smell: structuring the molecular odor space using semantic taxonomies and machine learning

**DOI:** 10.1093/chemse/bjag020

**Published:** 2026-07-08

**Authors:** Akshay Sajan, Stijn Sluis, Reza Haydarlou, Sanne Abeln, Pasquale Lisena, Raphaël Troncy, Caro Verbeek, Inger Leemans, Halima Mouhib

**Affiliations:** Department of Computer Science, VU Bioinformatics Group, Vrije Universiteit Amsterdam, De Boelelaan 1105, Amsterdam 1081 HV, The Netherlands; Department of Computer Science, VU Bioinformatics Group, Vrije Universiteit Amsterdam, De Boelelaan 1105, Amsterdam 1081 HV, The Netherlands; Department of Computer Science, VU Bioinformatics Group, Vrije Universiteit Amsterdam, De Boelelaan 1105, Amsterdam 1081 HV, The Netherlands; Department of Computer Science, AI Technology for Life, Universiteit Utrecht, Heidelberglaan 8, Utrecht 3584 CS, The Netherlands; EURECOM, Campus SophiaTech, 450 Route des Chappes, Biot 06410, France; EURECOM, Campus SophiaTech, 450 Route des Chappes, Biot 06410, France; Faculty of Humanities, Art and Culture, History, Antiquity, De Boelelaan 1105, Amsterdam 1081 HV, The Netherlands; KNAW Humanities Cluster, Oudezijds Achterburgwal 185, Amsterdam 1012 DK, The Netherlands; Department of Art and Culture, History, and Antiquity, Faculty of Social Sciences and Humanities, Vrije Universiteit Amsterdam, De Boelelaan 1105, Amsterdam 1081 HV, The Netherlands; Department of Computer Science, VU Bioinformatics Group, Vrije Universiteit Amsterdam, De Boelelaan 1105, Amsterdam 1081 HV, The Netherlands

**Keywords:** descriptor based odor taxonomies, machine learning for olfaction, open access odorant datasets, pear and fruity odorants, structure-based odor prediction

## Abstract

One of the key challenges to predict odor from the molecular structure is unarguably our limited understanding of the odor space and the complexity of the underlying structure–odor relationships. Here, we introduce an expert-curated taxonomy (ET) that captures the hierarchical relations between odor descriptors for molecular datasets. To quantify the usefulness and relevance of this expert taxonomy, we provide a systematic validation that leverages the predictive performance of machine learning models for structure-based odor predictions, as well as known structure–odor relationships. As a control next to the ET based on semantic and perceptual similarities, we provide a data-driven taxonomy (DT) based on clustering co-occurrence patterns of odor descriptors from our expert-curated molecular dataset. The latter is derived from available datasets in the Pyrfume repository. Both taxonomies (ET and DT) add value in the semantic organization of odor descriptors and provide an avenue for novel insights in molecular structure–odor prediction. Together with in-depth validation steps that highlight the value of odor taxonomies, the quality of the ET is quantitatively assessed. The DT further allows critical evaluation of the expert taxonomy, identification of potential inconsistencies, and a better understanding of the molecular odor space. Finally, we highlight the results of the expert-curated taxonomy by showcasing odor predictions for the case of pear odorants used in perfumery. Both taxonomies as well as a full molecular dataset are made available to the community, providing a stepping stone for a future community-driven exploration of the molecular basis of smell.

## 1. Introduction

**Figure 1:**
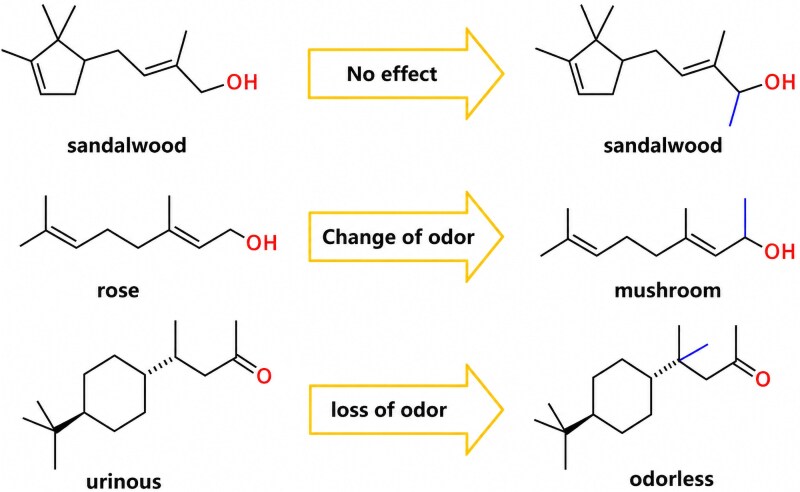
Impact of a small structural modification (addition of a single methyl group, symbolized by the blue line). The effect ranges from no change to a complete loss of odor. Examples taken from Sell (2006).

Despite numerous rules of thumb proposed by fragrance chemists over the years, predicting the smell of molecules directly from molecular structure remains a challenging task (Ohloff et al. 2012). One of the main goals in fragrance chemistry is to relate the physical and structural properties of odorants to their perceptual odor properties in order to rationally design new compounds of a desired smell. The complexity of predicting smell directly from structure arises from the fact that molecules with similar structures can possess very distinct odors, while structurally unrelated molecules with different structures may produce close to identical odors (Sell 2006). Figure 1 shows selected examples of odorant molecules (odorants) and their reported odor descriptors. In the different cases, it can be seen that the addition of a simple methyl group (depicted in blue) has different effects on the smell of the original odorant, ranging from no effect to a complete loss of the biological activity.

In addition to this and the complex underlying biology, describing a smell and quantifying it directly depends on individual perception of trained experts. This is a different situation from other senses such as vision and hearing that do not necessitate subjective input. Nonetheless, the overall complexity of the sense of smell provides a unique opportunity to explore the usefulness of machine learning (ML) techniques, for instance to identify relevant structural patterns directly from available molecular data without the necessity to elucidate the complex biology. While several machine learning approaches have been reported for this purpose over the past decade (Keller et al. 2017; Chacko et al. 2020; Sanchez-Lengeling et al. 2023), the most recent breakthrough was achieved using a graph neural network (GNN) to generate an embedding for molecular representations which is plotted as Principal Odor Map (POM) (Lee et al. 2023). Using this GNN-derived embedding space allowed for accurate structure-based odor predictions of novel odorants that outperformed median human panelists. The POM additionally validates the underlying perceptual odor similarity between odor descriptors and suggests an intrinsic conceptual hierarchy between the descriptors that may be explored by building a taxonomy of odor descriptors from molecular datasets. Although simple taxonomies that leverage odor similarities, based on clustering of odor descriptors using their co-occurrence from structure–odor data, have been reported before (Liu et al. 2019), these rely mainly on web scraping. As of today, there is no available validated open-source taxonomy for molecular descriptors that can be used as a separate machine learning task for interpretability of categorically higher-level umbrella terms. In this work, we address this gap and manually derive and validate an expert-curated taxonomy for molecular odor descriptors based on a selection of open access datasets available on the Pyrfume repository (Castro et al. 2022). This repository provides an agglomeration of several datasets with over 20,000 odorants and over 770 distinct odor descriptors. Still, currently available taxonomies do not focus on odor descriptors for molecules and are indeed lexica rather than taxonomies, i.e. they do not cover existing hierarchical relationships between odor descriptors. A taxonomy related to odor descriptors that can be found online but does not have the same purpose as the expert-curated taxonomy provided in this work is the *Osmo Scent taxonomy*, which, although not linked to molecules, provides a qualitative hierarchy that organizes 159 odor descriptors based on traditional perfumer practices. Additionally, some data-driven approaches have been reported before, that yielded smell lexica without hierarchical relations that further lack expert validation (Liu et al. 2019). Although there are also other olfaction related taxonomies for very particular applications, such as wine, whisky, or food flavors, these are not relevant to the field of structure–odor prediction and the therein defined machine learnable tasks which are one of the applications provided in this work. Therefore, we screened the different datasets from the Pyrfume database and selected datasets based on their reliability and usefulness (see methods section for details) and used 617 of the therein reported odor descriptors to compile a detailed multilayer expert taxonomy for molecular datasets. This hierarchical expert-curated taxonomy addresses an important gap in the field as there are no existing expert-curated taxonomies openly available to capture the hierarchical relations between odor descriptors in molecular datasets.

**Figure 2:**
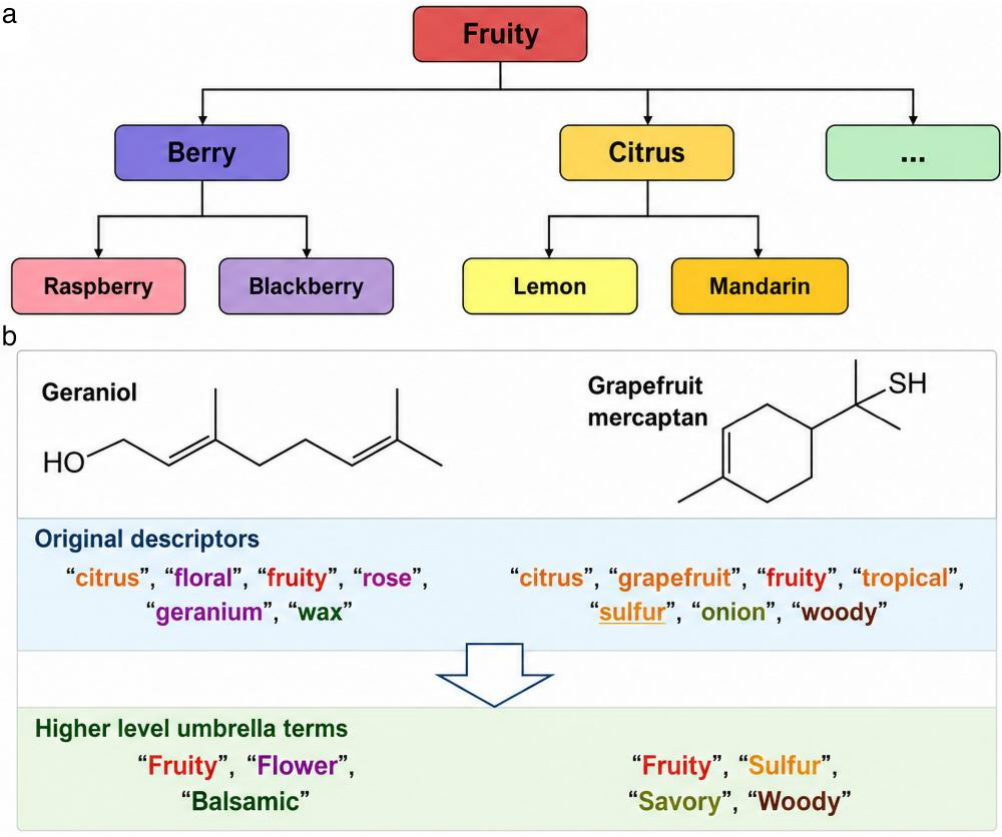
a) Visualization of the hierarchical relations between odor descriptors as provided in the expert taxonomy (ET). b) Example of applying one level of the expert taxonomy on the odor descriptors of the molecules geraniol and grapefruit mercaptan in the molecular dataset (MMD—see Methods section below). By introducing the first level of the expert taxonomy, the output space is reduced from six to three and seven to four descriptors for geraniol and grapefruit mercaptan for the subsequent machine learning tasks, respectively. The taxonomy is imposed upon the dataset by replacing the odor descriptor annotations of each molecule with their respective higher-level umbrella terms (descriptors falling under a higher-level umbrella term are color coded with matching colors).

In this work, we further investigate whether the hierarchical ordering of odor descriptors in a taxonomy helps convey essential information that allows us to deepen our understanding of structure–odor relationships. We provide three main deliverables, namely (i) a compiled molecular dataset for structure-based odor prediction tasks; (ii) an expert-curated taxonomy for odor descriptors that can be imposed for a novel machine learning task; and (iii) insight into model prediction and interpretability by enabling the analysis of molecular odor patterns in relation to higher-level odor categories. All the data, taxonomies, and models are openly accessible on the github repository (see Section 6). We evaluate the quality of our crafted expert taxonomy based on various validation criteria and conduct an interpretability analysis that reveals the underlying complexity of structure–odor relationships. Figure 2a illustrates the hierarchical structure of the expert taxonomy, and Fig. 2b illustrates how it is imposed on molecular odor annotations of two example molecules, geraniol and grapefruit mercaptan, to systematically reorganize the output space. Thus, the expert taxonomy organizes the existing odor space in a structured way that can be leveraged for improved interpretation and hypothesis generation. Note that in the case of grapefruit mercaptan the smell impression gradually shifts from "Fruity" to "Sulfur" within increasing concentration. This dosage-dependent effect is not taken into account in this work.

**Figure 3:**
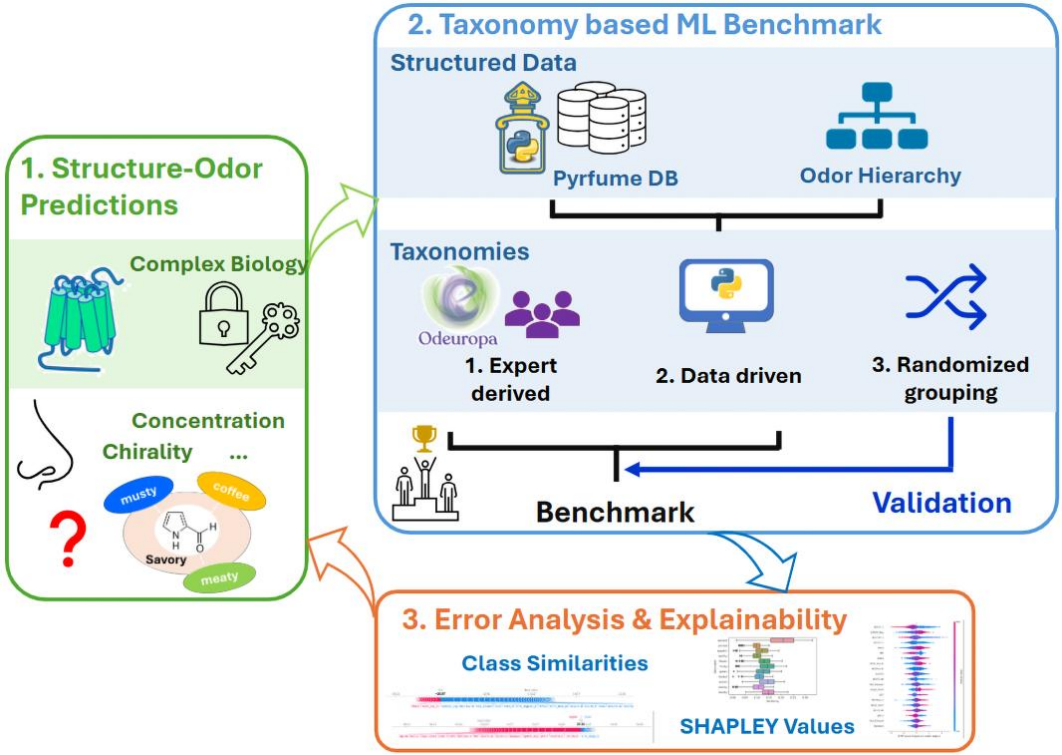
Schematic overview of the implementation used in this work. Expert and data-driven taxonomies are leveraged for structure–odor prediction tasks using machine learning to better understand structure-based odor predictions. Note that the data-driven taxonomy mainly serves as an additional way to assess the expert-curated taxonomy. We use randomized grouping of the 146 unique odor descriptors from the molecular dataset to benchmark the alignment of the two taxonomies to the structure of the odor space. Subsequently, the predictive performance of both taxonomies across different odor classes serves as a quality metric that allows for an in-depth error analysis which highlights the inherent complexity of structure–odor relationships.

A schematic overview of the overall workflow and conceptual motivation of this study is depicted in Fig. 3. Expert-curated and data-driven taxonomies are integrated into the machine learning pipeline, not with the goal of maximizing predictive performance, but to introduce a structured, human-interpretable organization into the existing odor space. By comparing expert-curated, data-driven, and randomized groupings across identical datasets, we assess the added value of the imposed hierarchical structure beyond raw labels via the expert-curated taxonomy. The resulting predictive performance across odor classes is then used as a diagnostic tool for taxonomy guided error analysis, enabling systematic examination of where structure–odor relationships are coherent, ambiguous, or inherently complex.

Finally, we discuss current limitations, challenges, and future opportunities and directions of structure-based odor predictions using machine learning. It should be noted that although there are more aspects of the olfactory sense where data have been gathered to leverage machine learning techniques, for instance in the physiology of odor recognition or genetic patterns in olfactory phenotypes, our work and the provided taxonomies circle around the task of structure-based odor prediction. For other purposes, the interested reader may consult several reviews that have treated these topics (Lötsch et al. 2019; Hao et al. 2025).

## 2. Methods

In order to structure the molecular odor space to leverage conceptual hierarchies between odor descriptors and deepen our understanding of molecular structure–odor relationships, we built a curated structure–odor dataset using the openly accessible Pyrfume database. The dataset is made openly accessible and that can be directly used for machine learning based prediction tasks. Using the existing odor descriptors from the Pyrfume database, we manually derived the first openly accessible expert-curated taxonomy for molecules. To assess the quality and usefulness of this taxonomy, we established a systematic validation pipeline that combines an additional data-driven taxonomy, controlled baseline comparisons (including randomized and pruned label groupings), and the behavior of both simple and high-capacity machine learning models (including the state-of-the-art OpenPOM model) next to known structure–odor relationships from fragrance chemistry. It should be noted that the modeling approaches are used as diagnostic and validation tools to evaluate the imposed structure of our taxonomies on the odor space.

### 2.1 Molecular dataset preparation

The molecular datasets used in this study were assembled using the open access repository Pyrfume. Pyrfume is an open-source project aimed at analyzing odorants and their features, providing both tools and access to diverse datasets. These datasets include molecular structures, perceptual data, and physicochemical properties, making it a valuable resource for researchers in the field. We use a selection of several datasets available on the Pyrfume repository (Castro et al. 2022) that comprises more than 40 stimulus-linked olfactory datasets across different species. In total, Pyrfume provides a selection of over 20,000 odorants and over 770 distinct odor descriptors. However, the repository also contains data that was obtained through web scraping and needs to be carefully checked before usage. For this work, we selected all the useful datasets from the Pyrfume collection based on domain knowledge and aggregated a molecular dataset that can be used to explore structure-based odor prediction using machine learning models.

From around 50 datasets in the repository, 14 datasets contained data about odor descriptors and compounds: Arctander (Arctander 1960), aromaDB (Kumar et al. 2018), Dravnieks (Dravnieks 1992), FooDB (Alberta Food Metabolome Project, led by David S. Wishart lab, n.d.), FlavorDB (Garg et al. 2018), Flavornet (Arn and Acree 1998), The Good Scents Company (n.d.), IFRA (“IFRA Fragrance Ingredient Glossary,” 2024), Keller (Keller et al. 2012, 2017; Keller and Vosshall 2016), National Geographic's Smell Survey (Wysocki and Gilbert 1989), Sharma (Sharma et al. 2021, 2022), Sigma Fragrance and Flavor Catalog (2014) (Sigma-Aldrich 2014.), Snitz (Snitz et al. 2019) and Leffingwell (Sanchez-Lengeling et al. 2020).

Among these, 7 datasets were selected to be used for the classification task: Arctander, aromaDB, FlavorDB, Flavornet, Goodscents, IFRA, and Leffingwell. The selection was based on: (1) datasets that concern human subjects (excluding animal based datasets) and (2) datasets that contain specific odor descriptors (unlike the rate-all-that-apply approach taken by the Dravnieks dataset) for single odorants (molecules, not mixtures). Furthermore, these are the most relevant datasets with respect to the reliability of the reported odorants, their odor descriptors, and listed physicochemical properties. The 7 Pyrfume datasets were merged and processed to create a comprehensive dataset with 6711 molecules and 146 distinct odor descriptors (see also Supplementary Figure S1 for details). It should be noted that this assembled dataset is curated by chemistry experts and can therefore be directly used for machine learning tasks without additional cleaning and processing steps. In the remainder of this paper, we will refer to this aggregation as Merged Molecular Dataset (MMD). Supplementary Figure S1 in the ESI depicts the full process of the expert-curated taxonomy derivation and the curation of the MMD. Table 1 provides a summary of the merged datasets, the publication year, the number of chemical compounds, and the number of odor descriptors.

**Table 1:** Summary of the datasets retrieved from the Pyrfume repository to (i) build the molecular dataset (comprising a total of 6,711 different molecules with 146 unique odor descriptors) used to train different ML classifiers and (ii) merge a maximum number of odor descriptors for the open access expert taxonomy, which now comprises a total of 557 unique odor descriptors and 60 hedonic odor qualities (see Section 2.2.1 for additional details).

Dataset	Publication year	No. of compounds	No. of odor descriptors
Arctander (Arctander 1960)	1960	2,580	762
AromaDB (Kumar et al. 2018)	2018	869	127
FlavorDB (Garg et al. 2018)	2018	525	255
Flavornet (Arn and Acree 1998)	2004	716	195
The Good Scents Company (n.d.)	2004	4,622	667
IFRA (“IFRA Fragrance Ingredient Glossary,” 2024)	2019	1,146	191
Leffingwell (Sanchez-Lengeling et al. 2020)	2001	3,487	113
MMD^a,b^	This work	6,711	146

^a^The number of compounds is not an exact addition over all datasets due to the overlap of samples that needed to be removed to avoid duplicates. Henceforward, this aggregation is referred to as Merged molecular dataset (MMD).

^b^Note that the data-driven taxonomy (DT) is based on the 146 odor descriptors of the MMD, while the expert-curated taxonomy (ET) comprises 617 odor descriptors from selected data sources of the Pyrfume repository.

Selected results from the initial data exploration, e.g. distribution of molecular weights, number of odor descriptors per molecular sample, and general statistics of the dataset are provided in Supplementary Figures S7–S10. The majority of the compounds possess 1–5 odor descriptors, while 32 compounds exhibit 13 or more descriptors, with a maximum of 17 descriptors. It should be noted that for each molecule, the strength of the individual odor descriptors is not provided, and the descriptors are therefore considered to be equally strong. In addition, the data is imbalanced toward “nice” smells that are linked to the food and perfume industry. There are more instances within the "Fruity" class and "Floral" class of the training dataset, 1,982 and 1,410, respectively, than in the camphor class with only 215 molecular samples.

To train and validate the expert taxonomy using machine learning models (see Section 2.3 for details), the dataset was split into a training and test dataset using a second-order iterative stratification (Szymański and Kajdanowicz 2017) to take the higher-order relationships between labels into account when doing a data split and make sure that the distribution of label pairs between splits stays consistent. The class distribution of the datasets is consistent across splits and is the case as well after imposing both taxonomies as can be seen in Supplementary Figures S15 and S16.

### 2.2 Manual expert-curated taxonomy derivation (ET) for molecular datasets and molecular odor predictions

To leverage the hierarchical connections within the odor descriptors for the machine learning classification, we rely on two different approaches: benefiting from expert domain knowledge by going through all 617 odor descriptors across the 14 previously mentioned datasets in Pyrfume and manually grouping them into classes that reflect existing relations between them, as well as generating a data-driven co-occurrence based clustering directly from the MMD. The detailed process to compile both taxonomies is described below.

In order to have a complete list of all odor descriptors, we aggregated descriptors coming from all 14 datasets if they had an identical label or if the Levenshtein distance between labels was <1. This enabled us to consider as a single concept small differences in spelling like “fish” and “fishy” or “wine-like” and “winelike.” The aggregations have been manually validated by experts to avoid any possible mismatch. This aggregation leads to 617 distinct descriptors. Most of these descriptors are present in only one source dataset.

Our team possesses expertise in chemistry, perfumery and historical smell culture, which allows us to propose a taxonomy to group the gathered 617 odor descriptors in different classes based on our domain knowledge. We decided to indicate descriptor groups: (i) source-based descriptors: descriptors relating the smell to a single odor (lemonlike, yeasty, wormwood) or overarching class of scents (fruity, woody, green) (557 concepts) and (ii) olfactory qualities: general adjectives expressing hedonic, trigeminal or emotional responses, or intensifiers (nice, fresh, fragrant, putrid, dry, light, heavy) (60 concepts).

The source-based descriptors were divided in 16 different classes “scent families” and 31 subclasses, e.g. the “Alcohol” class contains the subclasses “acid” and “alcohol,” which each contain their own odor descriptors. The list of classes and subclasses are provided in the Supplementary Information, the hierarchical expert taxonomy is provided on the ODEUROPA website https://vocab.odeuropa.eu/en/.

Henceforward, the taxonomy will be referred to as Expert-curated Taxonomy (ET). For this paper, we only consider the 557 source-based descriptors since the quality descriptors are harder to relate to specific odorant molecules. General statistics of the dataset after imposing the expert dataset are provided as Supplementary Information (See Supplementary Figures S13 and S14).

### 2.3 Systematic validation of the expert-curated taxonomy (ET)

To quantify the usefulness of the curated ET and to identify how well it reflects the structure and relationship between odor descriptors within the molecular odor space, several validation steps are put in place. Next to the ET, it is useful to generate a data-driven taxonomy directly from co-occurrence of odor descriptors within the MMD. This helps identify potential expert bias and can be used to further refine the ET. When a taxonomy is imposed on the dataset by replacing odor descriptors with their corresponding umbrella terms, the output space on which the machine learning model is trained is reduced from individual descriptors to higher-level categories. This reduction simplifies the learning task by increasing the amount of data per class and lowering the likelihood of misclassification. Consequently, performance should be expected to improve even when odor descriptors are grouped at random. Therefore, it is necessary to evaluate whether the performance achieved by the models trained on the ET imposed datasets exceed those explained solely by output-space reduction. To this end, the performance of the ET is compared against randomized groupings, as well as against a data-driven taxonomy (see section below). In this context, we interpret an improved performance as indicating a stronger signal and, as a consequence, a more coherent taxonomy structure. In addition, we compare the MMD after pruning labels against an undersampled ET to decouple the effects of output-space reduction and to set up a baseline. The pruning protocol and performance is provided in Supplementary Table S9.

#### 2.3.1 Data-driven taxonomy (DT)

**Figure 4:**
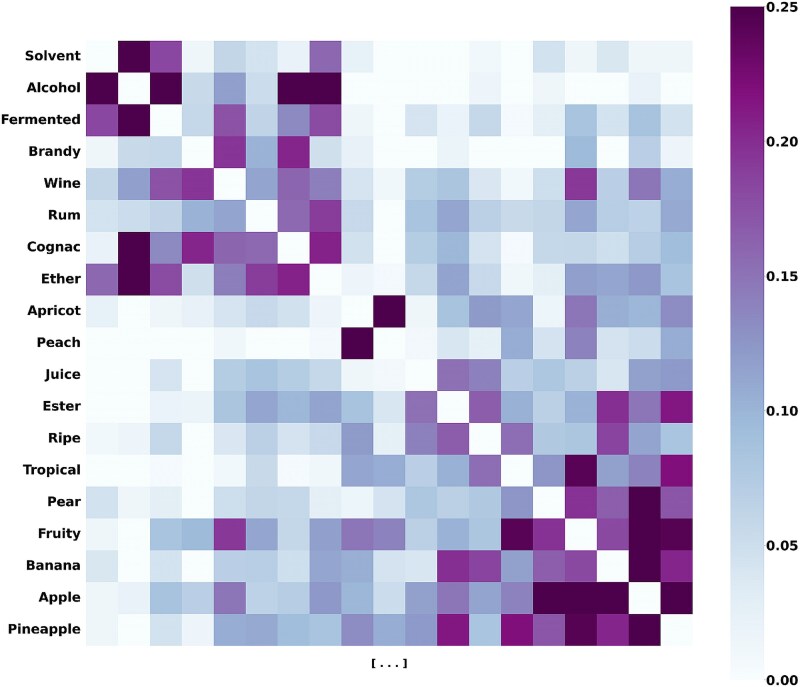
Selected subsection of the correlation matrix based on the co-occurrence of odor descriptors of the MMD. The full correlation matrix is provided as Supplementary Information (Supplementary Figure S6). The indices are sorted based on the clustering made using hierarchical agglomerative clustering with “ward” linkage and using Euclidean distances. A cluster of labels that often co-occur appears as a dark area on the heatmap. The data-driven taxonomy leverages these co-occurrence patterns to generate the classes and group the descriptors based on the occurrence throughout the data (see Methods section for additional details).

In order to generate an automated and data-driven taxonomy as an additional comparison to the ET, the co-occurrence patterns of the odor descriptors in the MMD were leveraged as they frequently co-occur with other perceptually similar odor descriptors. Therefore, a correlation matrix based on this co-occurrence was used to cluster the odor descriptors via agglomerative hierarchical clustering, a clustering method that iteratively merges individual descriptors to form groups with minimal internal distances, Euclidean in our case, as well as using Ward linkage to minimize inter-cluster variance (see Supplementary Information for parameters used). The elbow method suggests that the ideal cluster number is 43; however, the number of clusters was set to 16 to allow a fair comparison with the ET, where 16 classes were defined by experts to group conceptually related odor descriptors. Figure 4 depicts a zoom into 19 odor descriptors of the correlation matrix, based on the co-occurrence of the odor descriptors within the training split of MMD. The figure shows two main clusters that can intuitively be attributed to an “Alcohol” and a “Fruity” odor cluster. Note that the odor descriptors “Wine” and “Fruity,” as well as “Apricot” and “Peach” have a high frequency of co-occurrence, which would also be an intuitive association to most people. The full correlation matrix containing all 146 descriptors is provided as Supplementary Information (Supplementary Figure S6). Henceforward, this taxonomy will be referred to as Data-driven Taxonomy (DT). The complete list of all clusters is provided in the Supplementary Materials. In the discussion (see Section 3.2), the 16 classes from both taxonomies are compared against one another to elucidate the choices made by the experts compared to the clusters revealed directly from the co-occurrence matrix of odor descriptors (see also Supplementary Figures S11 and S12 for general statistics of the dataset after imposing DT).

#### 2.3.2 Machine learning models, feature selection, and hyperparameter tuning as validation framework for the expert-curated taxonomy

To evaluate whether imposing meaningful taxonomies is useful for gaining additional insight from molecular structure odor prediction by leveraging machine learning tasks, we evaluate the performance of OpenPOM (Barsainyan et al. 2023), the open-source reproduction of the principal odor map model, trained and tested on the MMD as well as both taxonomies (ET and DT). While OpenPOM is the state-of-the-art model, it still lacks interpretability to aid in the conceptual understanding of the molecular structure–prediction task. Therefore, three interpretable machine learning models were selected to benchmark the usefulness of taxonomies for structure-based odor predictions: Logistic Regression (LR), Random Forest (RF), and XGBoost (Chen and Guestrin 2016). For OpenPOM, the provided open access repositories by third party implementations were used for hyperparameter tuning, using random search cross-validation, as well as to determine the best ensemble number. The optimized hyperparameters for OpenPOM trained on MMD, DT, and ET are provided in Supplementary Tables S6–S8. For the other machine learning algorithms, the hyperparameter tuning of the models was done using Bayesian optimization through skopt (Head et al. 2020). The final features and hyperparameters used are provided in the Supplementary Information (Supplementary Tables S1–S3). Modred, a free open-source molecular descriptor calculator (Moriwaki et al. 2018), was used to generate molecular features from the Simplified Molecular Input Line Entry System (SMILES) of all the molecules in our datasets. The resulting molecular descriptors consist of a total of 713 features, after removing columns with missing values, ranging from atom types, number of specific atom types, acid/base counts, as well as older obscure chemoinformatic descriptors like Burden matrices and Chi-descriptors. The derived molecular descriptors consist of features using topological representations (2D) and geometrical representations (3D). The generated features then underwent an exhaustive feature selection procedure. Zero variance features were removed and then ANOVA F-values were used to filter the features and the best feature for each of the 146 classes was selected. This was then narrowed down further to 23, through recursive feature elimination, using a Random Forest with Permutation Feature Importance.

#### 2.3.3 Randomized grouping

In order to validate our approach and benchmark the predictive performance of the proposed taxonomies, we generated 1,000 randomized groupings of all 146 odor descriptors to provide taxonomies that group odors randomly, independently from any conceptual similarities. The randomizations were carried for the ET and DT. Each randomized group contains 16 classes and each class contains one or more random odor descriptors out of the 146 odor descriptors. The randomization sampling has been limited to 1,000 groups because permuting all 146 descriptors within their respective randomized groups yields 146 factorial (146!) possible combinations, which is not computationally feasible (see Supplementary Figures S3–S5). For each of 1,000 randomized groupings, the performance of the machine learning models is compared through the following metrics: macro average AUROC, F1, precision, and recall. Note that this validation step is crucial to show that the performance is not due to reducing the complexity of the classification task, but because the model is able to catch the semantic links among odor descriptors (see Supplementary Tables S12 and S13). We used this validation method for validating both expert- and data-driven taxonomies.

#### 2.3.4 Feature importance analysis for evaluating the expert-curated taxonomy

To investigate the most relevant features that the machine learning model uses for its predictions, we investigated the feature importance of the best performing interpretable model from each task. First, the Permutation Feature Importance (PFI) was used to assess the impact of each feature on the model performance. Here, permutations are added randomly to the features of the test data to rank features by measuring the decrease of a given score metric. A key limitation of PFI is the lack of class-specific insight it can provide as it focuses mainly on the overall macro scores. To address this, we subsequently carried out a SHapley Additive exPlanation (SHAP) value analysis (Lundberg and Lee 2017). This method makes use of cooperative game-theory to assign a value to each feature representing its contribution to the classification decision of the model. The SHAP values are plotted in summary plots for each class to understand the feature contributions for each class for both taxonomies to interpret the model output and the decision making that went behind it. It should be noted that this section does not directly aim to identify novel biochemical insights, but rather to verify if known relationships allow us to demonstrate how the expert-curated hierarchical taxonomy helps organize and explain model behavior at the category level. This additionally helps to see whether the ET adds an essential layer of semantic organization that is missing from the flat all descriptor list and still allows known relationships to be viewed coherently across related odor groups. See Supplementary Figure S2 for a detailed schematic workflow of the machine learning pipeline.

## 3. Results and discussion

### 3.1 Validation of taxonomy-based tasks using OpenPOM

To assess whether imposing odor taxonomies on molecular datasets yields valid machine learning tasks, both the expert-curated taxonomy (ET) and the data-driven taxonomy (DT) were imposed on the MMD and trained using the available OpenPOM model. These results are summarized in Table 2, including the optimized hyperparameters reported in Supplementary Tables S6–S8. As shown in Table 2, both the taxonomy imposed datasets achieve AUROC values over 0.8 with OpenPOM for multilabel classification. Overall, these results demonstrate that imposing ET and DT preserves meaningful signal and class structure, confirming that the proposed taxonomy-based representations constitute valid and informative machine learning tasks.

**Table 2:** Performance metrics of the OpenPOM model trained and tested on the MMD as well as the ET and DT imposed datasets.

Dataset	AUROC	F1 score	Precision	Recall
MMD	0.877	0.185	0.269	0.176
ET	0.838	0.549	0.535	0.596
DT	0.854	0.561	0.565	0.593

### 3.2 Taxonomy validation through randomized grouping of odor descriptors

**Figure 5:**
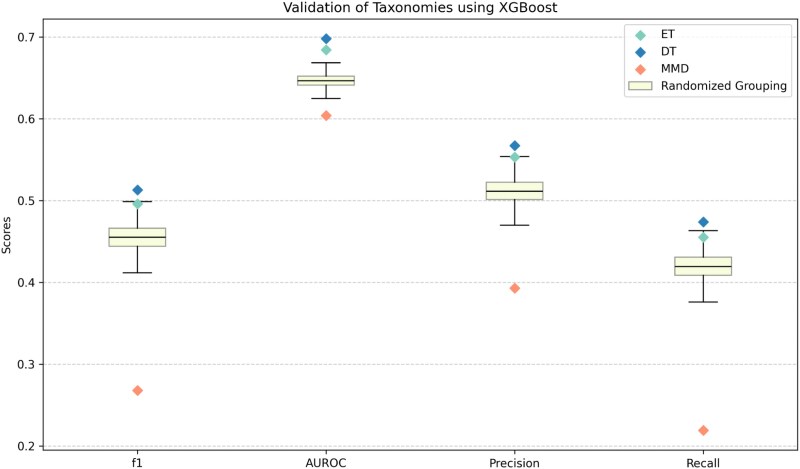
Box plot comparing the performance metrics of the XGBoost classifier using randomized groupings, the two taxonomies (ET and DT), and the original descriptors. The numerical values of each metric are provided in Table 3.

For comparison with the randomized grouping as well as for interpretability, we benchmarked simpler machine learning models such as LR, RF, and XGBoost. Of these, XGBoost had the best performance and as such, we selected it to compare the machine learning tasks derived from randomized grouping. The results of the LR and RF models, as well as the class-wise scores of the XGBoost model for both taxonomies are provided in the ESI (Supplementary Tables S4–S5 and S10–S11). To evaluate the predictions using the two structured taxonomies, ET as well as DT, we first established a baseline model using the full unstructured set of odor descriptors. This initial step allows us to assess the inherent difficulty of predicting individual odors directly from molecular features using simple machine learning models and to contextualize the benefit of using structured hierarchical taxonomies. The baseline model (an XGBoost classifier), trained to perform multilabel classification over 146 classes (using the MMD), achieved an overall score of 0.6 AUC, which is above random and suggests that the model is able to capture some meaningful signal in the data. However, this performance remains modest indicating that this is not a trivial classification task. There are several potential limitations that can lead to this, such as difficult feature representation, the limited data quality, and potentially difficult class separability based on underlying structural patterns in the data. Despite extensive hyperparameter tuning, none of the models yielded performances as reported in previous preprint work (Sanchez-Lengeling et al. 2023). We thus used these model predictions as a baseline and compared them to the behavior of the models when the derived taxonomies are imposed and the classification task is reformulated to 16 classes. The comparative evaluation of the different models using an XGBoost classifier is provided in Table 3 and Fig. 5. The results show that models trained on the dataset with imposed taxonomies show more consistent behavior compared to models trained on the original odor descriptors across all metrics for all the classifiers. Both taxonomy-based models significantly differ from the models based on the randomized groupings of odor descriptors. It should be noted that the performance metrics of the randomized models are higher than 0.5, which would mean that the models are still picking up on a signal or structure in the data despite the loss of meaningful class groupings from the taxonomies. This is because the randomized groupings, for the most part, are not entirely meaningless due to the intercorrelation of many odor descriptors. This demonstrates the inherent complexity and interrelated nature of the odor descriptor space and the limitations of a two-layer taxonomy to capture conceptual links between different descriptors. Despite the high number of possible combinations for the randomized groupings (146!, i.e., 146 factorial in total), it is almost unavoidable to create classes where none of the descriptors are correlated. The scores for the randomized groupings can serve as a baseline reflecting the ability of the model to extract signal from noise and serve as a proxy for the structural coherence of ET and DT. Hereby, a higher score indicates a better conceptual clustering and arrangement of the odor descriptors. Both taxonomies show improvements over the random groupings, with the DT showing a marginal advantage over the ET.

**Table 3:** Performance metrics of the XGBoost classifier as obtained using the full 146 descriptors, the taxonomies, and the randomized grouping.

Dataset	AUROC	F1	Precision	Recall
MMD	0.604	0.268	0.393	0.219
ET^a^	0.684	0.496	0.553	0.455
DT^a^	0.698	0.513	0.567	0.474
Random grouping^b^	0.648 (07)	0.455 (15)	0.511 (15)	0.418 (15)

The results are visualized in Fig. 5. The corresponding performance metrics for the random forest and logistic regression models are provided as Supplementary Information.

^a^The class-wise performance metrics are provided in the ESI (Supplementary Table S10 and Supplementary Table S11).

^b^The standard deviation for the 1,000 randomized grouping is given in parentheses with respect to the last 2 digits (see ESI for additional details).

However, in order to show that introducing a hierarchical expert-curated odor taxonomy for molecules provides a meaningful and structured representation over raw labels, even using simple ML models, the improvement from 0.6 to almost 0.7 already indicates that the taxonomy adds a usable signal. The randomized groupings additionally support this claim and allow us to contextualize the role of the ET, showing how structuring the molecular odor space by leveraging existing hierarchies between odor descriptors influences ML tasks. While the aim of this work is not to compete with the latest state-of-art, the open-source reproduction of POM (“OpenPOM”) was additionally trained on the DT and ET taxonomies for the sake of completeness. The presented modeling experiments serve to illustrate a practical use case and provide a means to evaluate the imposed structure. Although the performance of the implemented ML models do not define the value of the taxonomy, the models enabled improved error analysis and interpretability, i.e. the identification of underlying molecular patterns that are relevant for structure–odor predictions. Interestingly, the ET (even collapsed to 2 levels and therefore resembling a lexicon—see also Fig. 2b) is able to capture existing links between the odor space and the underlying molecular structures.

### 3.3 Conceptual comparison of the expert-curated and data derived taxonomies

Table 3 showed that the DT yielded higher scores for the different metrics when compared to ET. This is a consequence of deriving the DT directly from the data itself. However, The DT is fundamentally constrained as a methodology to formulate odor taxonomies due to its reliance on the frequency of odor descriptors for co-occurrence clustering. This means that less frequently occurring descriptors are clustered together in conceptually meaningless clusters. Still, it is useful for our purpose to identify biases that might be introduced by experts when curating the ET. To provide insight into how well each taxonomy reflects meaningful relationships between odor descriptors and how these align with known concepts in perfumery and sensory science, it is essential to go beyond purely quantitative comparison and include semantic coherence, domain knowledge, and structural elements of the taxonomies. Therefore, we compare the 16 data-driven classes (A to P) to the 16 expert-curated classes to identify conceptual similarities between them. The complete list of odor descriptors and the different odors grouped together to build the taxonomies are provided in the Supplementary Information (see Supplementary Tables S12 and S13). While this comparison highlights where the two taxonomies overlap and differ from one another, it also raises the question of how easily each grouping of odor descriptors may be interpreted by a human reader or by a Large Language Model (LLM). To address this, we used the latest version of ChatGPT (GPT-4o) to define class names for the 16 classes within the DT (the prompt used to generate the classes is provided in the ESI). The comparison is shown in Table 4. While several classes of the DT can be directly mapped to the classes defined within the ET, i.e. “Gourmand,” “Flower,” “Alcohol,” “Savory,” “Fruity,” “Sulfur,” and “Woody,” the others are more difficult to assess. The classes that can be directly mapped to expert defined classes reflect groupings of odor descriptors that are mainly based on conceptual odor similarities, which was the central element to structure the ET. Other data-driven classes, e.g. class “E” cannot be directly mapped to one of the 16 expert defined classes. Class “E” contains a mix of conceptually related odor descriptors such as “cinnamon,” “clove,” “spicy,” and “vanilla” which may be grouped under the higher term “Spicy.” However, it also includes seemingly unrelated terms such as “balsamic,” “medicinal,” “phenol,” and “smoked.” These descriptors are conceptually not similar to the listed “Spicy” descriptors, but rather evoke medicinal practices or properties. Vanilla is also part of the "Gourmand" family within perfumery and sometimes classified as having a green aspect. The DT derived class “E” can therefore rather be described as a “Health” or “Medicinal” category. More in alignment with the ET that shows inherent bias toward grouping odor descriptors on conceptual similarities, the suggestion from the ChatGPT describes class “E” as “picy” without taking the outliers “balsamic,” “medicinal,” “phenol,” and “smoked” into account. It should be noted that “medicinal” and “smoky” do not seem source based, whereas the other descriptors do. Often myrrh, which is a resin, is qualified as medicinal (Batiha et al. 2023).

**Table 4:** Comparison of the 16 odor classes within the 2 defined taxonomies, including the number of descriptors within each class.

Class^a^	No. of descriptors^b^	Class^c^	No. of descriptors^d^	Similarity^e^	LLM^f^
Alcohol	10	A	12	Anethole	Aromatic
Animal	4	B	14	**Gourmand**	**Gourmand**
Aquatic	3	C	22	**Flower** ^g^	**Floral**
Balsamic	2	D	13	**Pungent**	**Pungent**
Chemical	12	E	8	Health/Medicinal^h^	Spicy
Earthy	5	F	8	**Dairy/Cream** ^h^	**Lactonic**
Flower	15	G	8	**Green**/Fat^h^	**Green**
Fruity	28	H	8	**Alcohol**	**Alcoholic**
Gourmand	13	I	7	**Savory**	Animalic
Green	9	J	11	**Fruity**	**Fruity**
Herbal	5	K	8	Fresh^h^	Citrus
Savory	23	L	6	Clean^h^	Camphorous
Smoky	5	M	4	**Acid**	**Acidic**
Spices	8	N	3	**Sulfur**	**Sulfurous**
Sulfur	3	O	10	**Woody**	**Woody**
Woody	9	P	4	**Berry**	**Berry**

The class definitions for the DT groupings (Classes A to P) are compared to the ET categories and to categories provided through a large language model (LLM: GPT-4). Class descriptions that are similar between the two taxonomies are highlighted in bold. The full list of descriptors within the different classes of the ET and DT are provided as Supplementary Information (Supplementary Tables S12 and S13).

^a^16 classes defined by domain experts for the expert-curated taxonomy.

^b^Number of descriptors included in each class of the expert-curated taxonomy.

^c^16 classes obtained from the data-driven taxonomy.

^d^Number of descriptors included in each class of the data-driven taxonomy.

^e^Conceptual similarity of the data-driven classes to the expert defined classes as estimated from the experts' perspectives.

^f^Suggested class name for the descriptors in the different classes of the data taxonomy as suggested using the large language model (LLM) ChatGPT-4o. See Supplementary Information for the list of descriptors within the different classes.

^g^In perfumery this class is usually called “Floral.”

^h^Note that not all the classes from data taxonomy can be mapped directly to classes within the expert-curated taxonomy. The highlighted classes are not fully based on conceptual similarities but also evoke effect or odor practices (see the text for more details).

There are four other DT derived classes that exhibit similar patterns: classes “F,” “G,” “K,” and “L.” Looking closer into the descriptors of class “K” and class “L,” the overarching name for these classes may be set to “Fresh” and “Clean,” while the LLM suggests “Citrus” and “Camphorous,” respectively. Here, we need to keep in mind that odor descriptors such as “clean” are often culturally determined and may be linked to different odors in different countries. Overall, our comparison shows that the DT does not solely rely on conceptually related odor classes but includes the effect and quality that the descriptors share in the human world. In contrast, the ET classifies descriptors more on the odor impression informed by pre-existing expectations from the side of the experts, their knowledge of chemistry, historical scent classifications and existing scent wheels used in perfumery (Ohloff et al. 2012). This is also reflected in the selection of the grouping terms generated by the LLM, which tries to flatten the different descriptors within the DT classes back to a lower dimension that is similarly biased toward conceptual odor similarities as the expert-curated classes. This represents a first step toward further refining ET and aligning it more closely with the molecular odor space.

### 3.4 Error analysis and interpretability of the classification results using XGBoost

**Figure 6:**
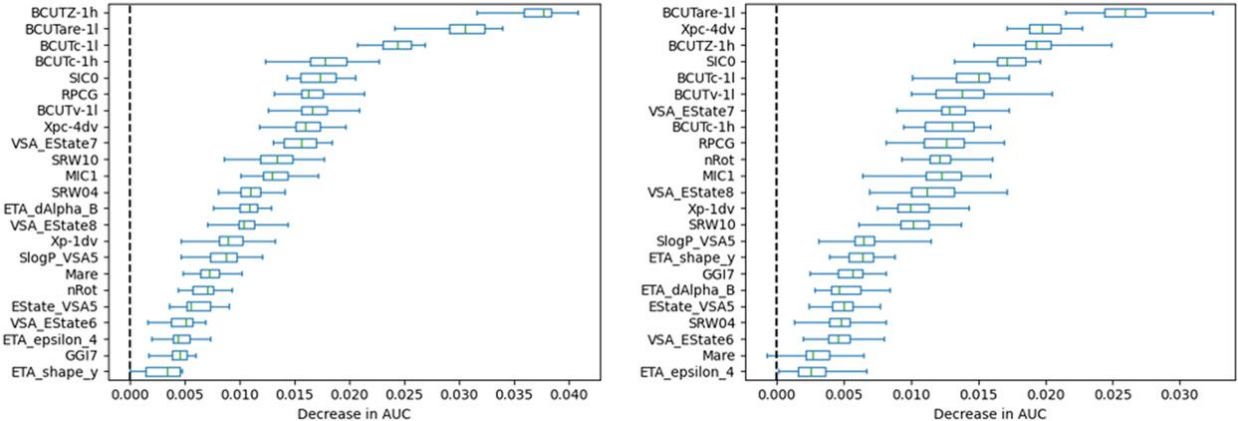
Permutation feature importance (PFI) of the XGBoost model for the data-driven taxonomy (left, DT) and the expert-curated taxonomy (right, ET). Features causing the largest decrease in macro-AUROC upon permutation were "BCUTZ-1h", "BCUTare-1l", and "BCUTc-1l" for the DT, and "BCUTZ-1h", "BCUTare-1l", and "Xpc-4dv" for the ET, respectively. Note that class-specific relevance needs to be taken into account to obtain more insight into the feature importance of the models.

To shed some light into the underlying structure–odor relationships of our dataset and taxonomies, it is important to conduct an error analysis on misclassifications of the model as well as interpretability studies such as Permutation Feature Importance (PFI). These insights help to verify that the model is learning meaningful patterns rather than artifacts and allow identifying limitations and bottlenecks to guide future feature engineering and model refinements. The PFI plots of the XGBoost classifier (the best performing model see Section 3.2) is provided in Fig. 6 for both taxonomies (see Supplementary Figures S17 and S18 for XGBoost feature importance). Notably, several features originating from the Burden matrix such as “BCUTZ_1h” and “BCUTare-1l” exhibit significant drops in the AUC upon permutation, indicating their strong influence on the model performance. However, it should be noted that PFI is more indicative of overall performance of the model and that the class-specific relevance still needs to be taken into account to gain deeper insight into the underlying feature importance of the model.

**Figure 7:**
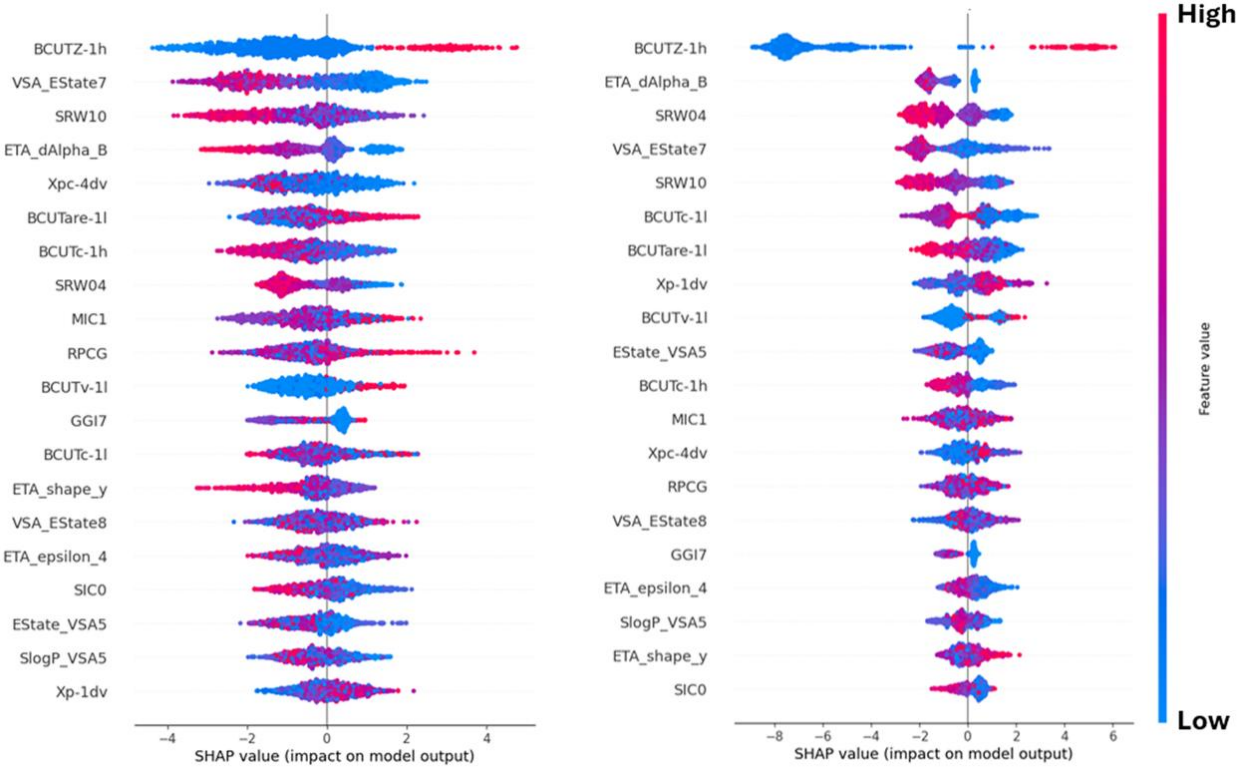
SHAP analysis of the XGBoost classifier for both taxonomies for the ET-defined “Sulfur” class (left) and the corresponding DT class (Class “N”) (right). We can see that the feature “BCUTZ-1h” is listed as the most important feature for classifying “Sulfur” across both taxonomies. For the full list of descriptors, see Supplementary Information.

**Figure 8:**
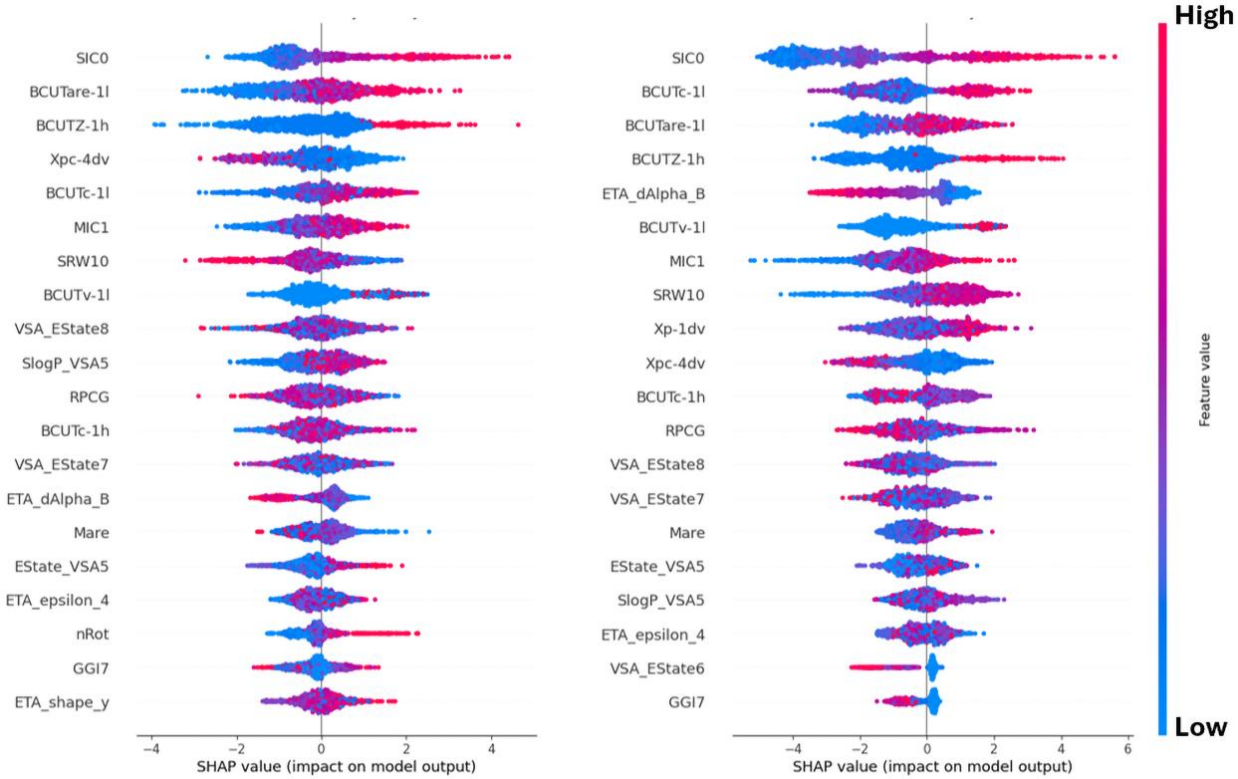
SHAP analysis of the XGBoost classifier for both taxonomies for the ET-defined “Savory” class (left) and the corresponding DT class (class “I”) (right). Note that in both taxonomies, the feature “SIC0” has the biggest contribution, overtaking “BCUTZ-1h” from the “Sulfur” class for both taxonomies.

Additionally, the SHAP plots for the "Sulfur" and "Savory" class can be seen in Figs. 7 and 8. The SHAP plots of all remaining classes for both taxonomies are provided in the ESI (Supplementary Figures S20 and S21). Altogether, the “Sulfur” and “Savory” classes are the best performing classes for both taxonomies with AUC over 0.75. The feature which contributes significantly to the “Sulfur” class across both taxonomies is “BCUTZ-1h,” which is particularly important within the SHAP values of the DT. The “BCUTZ-1h” feature represents the highest eigenvalue of the Burden matrix weighted by the atomic number. This corresponds to the atomic number of the heaviest atom in the compound, which is 16 in sulfur-containing compounds. This feature helps in classifying with both the “Sulfur” and “Savory” classes, which are both similar in odor profile (see Fig. 7). There is also minor variation in the SHAP values of the "Sulfur" class across both taxonomies as well, where in the DT, the influence of "BCUTZ-1h" is more pronounced as compared to the ET where it shares similar weight with other features. This might be indicative of the odor descriptors under their respective umbrella terms, wherein the “Sulfur” class in the DT is more narrow while that of the ET is a bit more general. We can see this in the distribution of "BCUTZ-1h" values across different taxonomies (see Supplementary Figure S19): where in the DT, both the “I” and “J” classes, “Savory” and “Sulfur,” respectively, have typically a higher "BCUTZ-1h" score with the "Sulfur" class being more pronounced, and in the ET, the distribution of "BCUTZ-1h" is more similar for “Savory,” “Smoky,” and “Sulfur.” It is interesting to note that there is a distinction between "Savory" and "Sulfur" and the features that help predict it. While "BCUTZ-1h" is the top feature for both taxonomies, when considering “Savory,” the feature "SIC0" seems to be the most important predictor across both taxonomies. “SIC0” is the structural information content of the molecule normalized by atom count. This feature corresponds to the degree of structural complexity where a high value suggests distinct atom types or diversity in atom degrees and a low value suggests more uniformity in atom types. The SHAP plots show that for the "Savory" class, a higher "SIC0" value pushes the model toward a positive classification for "Savory" as compared to “Sulfur” where interestingly, the inverse occur where for both taxonomies, a higher "SIC0" value pushes the model against classifying Sulfur. This could imply that minute variations in distinct atom types or diverse atom degrees could explain the minor differences in the odor profiles of both “Sulfur” and “Savory.” Finally, it should be noted that these two key structure–odor findings are well established in the literature and therefore do not directly reveal novel biochemical insight. However, in the context of this work, our taxonomy guided machine learning models clearly capture known relationships and demonstrate how the expert-curated hierarchical taxonomy helps organize and explain model behavior at the category level. Thus, the ET adds an essential layer of semantic organization that is missing from the flat all descriptor list and allows the relationships to be viewed coherently across related odor groups.

### 3.5 Testing the taxonomy models for class prediction: the case of pear odorants

To evaluate the applicability of the taxonomy models on interesting odorants used in perfumery, we selected four pear odorants and classified their odor descripors using the two XGBoost taxonomy-based models. Pear odorants play a well-established role in perfumery due to their popularity as a top note in perfumes where they are often used to design fresh, bright, and juicy fragrances.

**Figure 9:**
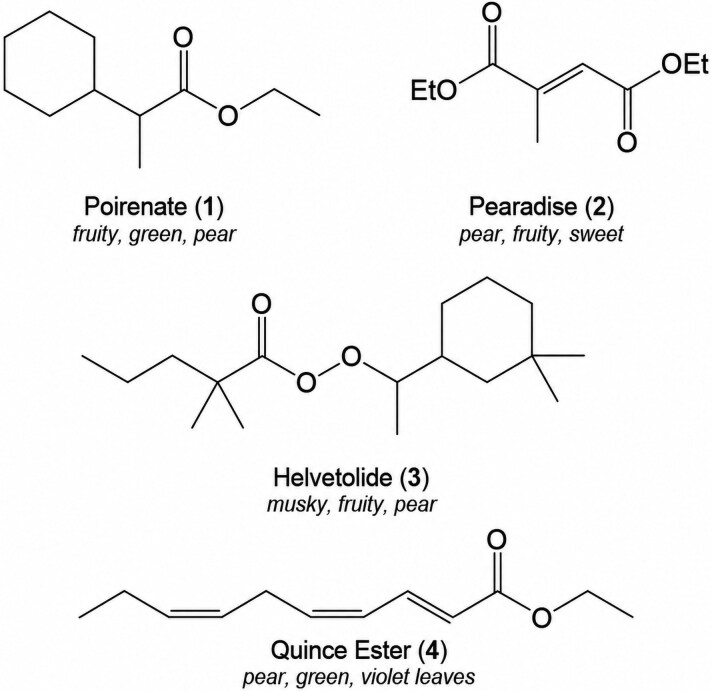
Selected examples of pear odorants. Note that all are correctly classified as “Fruity” using both taxonomies (See Table 5).

The molecular structures of the four pear odorant molecules used in perfumery, as well as their reported odor descriptors, are depicted in Fig. 9. It should be noted that Pearadise (**2**) is an artificial odorant that was rationally designed based on known structures of pear odorants with the aim to be fully biodegradable and sustainable (Elterlein et al. 2024). In future, next to specific odor and smell impressions, biodegradability and biocompatibility will become increasingly important and raise the challenge and requirements on machine learning methodologies within the fields of fragrance chemistry and in silico molecular design (Armanino et al. 2020).

The selected subset of odorants serves as domain specific test case to assess how well our models generalize beyond the training distribution. The selected odorants from this odor family is clearly related to the “Fruity” and the “J” classes of the ET and DT, respectively. The classifications results are shown in Table 5. Interestingly, both models are able to classify the most relevant odor descriptors of the pear odorants, namely, “Fruity” and “Green.” Going forward, it will be interesting to look into the explicit odor descriptors within the taxonomy groupings to distinguish more specific odors and continue to delineate the odor space and the structure–odor relationships linked to it.

**Table 5:** Classification results using the XGBoost model for the expert- and data-driven taxonomies for five selected pear odorants used as top notes in perfumery (see Fig. 9 for the corresponding chemical structures).

Pear odorants	Data-driven taxonomy^a^	Expert-curated taxonomy
Poirenate (1)	C, J, O	Animal Body, Flower, Fruity, Woody
Pearadise (2)	J	Fruity
Helvetolide (3)	C, G, J	Fruity, Green, Woody
Quinceester (4)	G, J	Fruity, Green

^a^Classes C, G, J, and O can be conceptually mapped to the expert classes "Flower", "Green"//Fat", "Fruity", and "Woody", respectively (see also Table 4 for a complete overview).

## 4. Conclusion

In this work, we provide an openly accessible, well curated, and structurally organized molecular odor dataset, and most importantly, an expert-curated taxonomy (ET) that groups odor descriptors into meaningful hierarchical groupings that can be used to train machine learning models. The novelty of presented work lies in directly addressing the gap of an expert-curated taxonomy for molecular odor descriptors and in providing a systematic approach to quantify the capability of this ET to capture the links between the molecular structure and the conceptual odor space in order to formalize the molecular odor space. Next to the expert taxonomy, an expert-curated merged molecular dataset (MMD), that can be directly used for odor prediction using machine learning, is made openly accessible to the community. The presented approach shows that perceptual odor taxonomies can be incorporated into molecular structure–odor datasets as a form of data augmentation, allowing us to shift the classification task from high to low granularity, thereby consolidating sparse and heterogeneous labels into structured, semantically coherent classes while preserving meaningful signal for machine learning tasks. Thus, the ET is intended as a structural and interpretive framework to improve our understanding of the complex odor space and underlying structure–odor relationships and does not aim to replace existing high-capacity deep learning models. The systematic benchmark using randomized groupings of odor descriptors showed that not all reductions of the output space are equally informative. In contrast, the provided taxonomies (ET and DT) based on meaningful groupings provide a more coherent and structured representation of odor space, enabling more consistent learning and interpretation. This allowed us to gain a deeper understanding of the underlying MMD dataset and, through the imposed structure, enabled the generation of new hypotheses on structure–odor relationships for future works, while contributing to the systematic organization of odor space. This study shows how multifaceted odor taxonomies can be. It compares our preconceived notions of distances between odor descriptors in the odor space (ET) with the DT which takes into account the individual shared experiences with odor that together shape the topology of the odor space. Together with interpretable decision-tree-based models, our study allowed us to link molecular structures to their respective odor space categories and pave the way for future machine learning approaches in structure-based odor prediction. The presented framework thus lays the foundation for more accurate, interpretable, and scalable approaches to decoding the language of smell and helps us move closer toward the digitization of one of our most enigmatic senses.

## 5. Outlook and perspectives

Currently, the most promising machine learning models for structure-based odor prediction are deep learning approaches, which have shown reliable performance in QSAR tasks (Chen et al. 2018; Sun et al. 2020). However, conventional deep learning methods do not offer much in terms of interpretability. Therefore, our approach goes back to simpler models which offer more interpretability to aid insight into structure–odor relationships by leveraging the conceptual hierarchy between smell. Moving forward, graph-based architectures, particularly more recent ones like Fragnet (Panapitiya et al. 2026) and KerGNNs (Feng et al. 2022), offer the potential for improved interpretability and more refined modeling of structure–activity relationships using deep learning. Odor taxonomies could be integrated into these GNNs not only to explore higher-level odor classes but also potentially within the loss function itself, guiding fine-grained classification across all descriptors. The ET, although explicitly flattened for this study, consists of multiple hierarchical layers that can be incorporated into the GNN architecture. Therefore, the hierarchical framework in this work paves the way toward a deeper mechanistic understanding in future works, i.e. by enabling more systematic analysis across and within odor families, guiding model architectures, and supporting the integration of perceptual, semantic, and structural signals. For the DT, the correlations between different odor descriptor groupings can also be further leveraged to create a multilayer hierarchical taxonomy. Such methods could pave the way toward a deeper understanding of structure–odor relationships and the rational design of novel odorant molecules. Despite the relevance of taxonomies for odor classification, there are several bottlenecks still limiting the progress that can be achieved in structure–odor prediction tasks. One important aspect is unarguably the 3D molecular structure of odorants. This work, as well as the current state-of-the-art rely on 2D molecular graphs as input, which omit conformational variability and spatial features that are likely relevant to odor perception. Odorants are inherently three-dimensional and can adopt multiple conformations, some of which may be more relevant to receptor binding and perceived odor than others. Incorporating 3D molecular geometry has already shown promise in related molecular classification tasks (Stärk et al. 2022), and methods such as PhysChem (Yang et al. 2021) that leverage molecular dynamics to represent conformations may extend this potential further. Still, receptor-level specificity adds another layer of complexity—olfactory receptors may respond differently to different conformers, but we currently lack sufficient experimental data to explore this (Mainland et al. 2015).

In future, research that focuses on receptor-odorant dynamics and mechanisms could be leveraged to make better datasets and consequently, more well informed machine learning models. This would also allow us to account for differences between enantiomers and explore the impact of chirality (Scott et al. 2022). Additionally, current datasets do not account for concentration-dependent effects and do not provide any information on the weight of odor descriptors, i.e. the dominant descriptors compared to less relevant side-notes. Concentration dependence is crucial, as the perceived odor of a molecule can vary with concentration. This effect is particularly strong for sulfur and nitrogen-containing compounds. To address these shortcomings, more high quality sensory data with different concentrations are required. Our work currently focuses on single odorant odor prediction without considering the effect of odorant mixture. In Olfaction however, smell sources emit hundreds of molecules that interact together to create a specific smell (Fricke and Schieberle 2020; Yu et al. 2023; Han et al. 2024). Therefore, to digitize smell and recreate odor compositions, it is necessary to move toward the study of odorant mixtures and concentrations, while keeping in mind that in some cases, olfactory groups and descriptors can additionally depend on social, religious, linguistic, or other culturally induced categories (Classen et al. 2002; Majid and Burenhult 2014).

## Supplementary Material

bjag020_Supplementary_Data

## Data Availability

The MMD, the taxonomies (ET and DT) including their taxonomy augmented datasets, and annotated code, for all the methodology as well as the analyses, are included as Supplementary material. In addition, all annotated code used for data processing, taxonomy construction, model training, and analysis is included to ensure full reproducibility of the results. The results of all benchmarking experiments, including the OpenPOM-based evaluation, randomized groupings, and pruning controls, together with all datasets, taxonomies, and code are publicly available on GitHub.
