# Supplementary material for: Hierarchies of smell: structuring the molecular odor space using semantic taxonomies and machine learning
Source: Chem Senses. 2026 Jul 8;51:bjag020. doi: 10.1093/chemse/bjag020 (PMC13415458; doi:10.1093/chemse/bjag020)
Supplement: bjag020_Supplementary_Data [file bjag020_supplementary_data.pdf]

Electronic Supporting information

Hierarchies of Smell: Structuring the molecular odor space using Semantic Taxonomies and machine learning

Akshay Sajan,[a] Stijn Sluis,[a] Reza Haydarlou,[a] Sanne Abeln,[b] Pasquale Lisena,[c] Raphael Troncy,[c] Caro Verbeek,[d] Inger Leemans,[e] and Halima Mouhib[a]*

[a] Department of Computer Science, VU Bioinformatics Group, Vrije Universiteit Amsterdam, De Boelelaan 1105, 1081 HV Amsterdam, The Netherlands.
[b] Department of Computer Science, AI Technology for Life, Universiteit Utrecht, Heidelberglaan 8, 3584 CS Utrecht, The Netherlands
[c] EURECOM, Campus Sophia Tech, 450 Route des Chappes, 06410 Biot, France.
[d] Faculty of Humanities, Art and Culture, History, Antiquity, De Boelelaan 1105, 1081 HV Amsterdam, The Netherlands
[e] KNAW Humanities Cluster, Oudezijds Achterburgwal 185, 1012 DK Amsterdam, The Netherlands. Vrije Universiteit Amsterdam, Department of Art and culture, History, and Antiquity, Faculty of Social Sciences and Humanities, De Boelelaan 1105, 1081 HV Amsterdam, The Netherlands.

ORCID:

Akshay Sajan	0009-0009-6777-0193
Stijn Sluis	0009-0001-0275-6317
Reza Haydarlou	0000-0003-4138-7179
Sanne Abeln	0000-0002-2779-7174
Pasquale Lisena	0000-0003-3094-5585
Raphael Troncy	0000-0003-0457-1436
Caro Verbeek	0000-0001-9691-5923
Inger Leemans	0000-0003-1640-4109
Halima Mouhib	0000-0001-5031-3468

*corresponding author: h.mouhib@vu.nl

Molecular odor descriptors for the provided molecular data set used to build the machine learning models (in alphabetical order).

['acid', 'alcohol', 'aldehyde', 'almond', 'amber', 'animal', 'anise', 'anisic', 'apple', 'apricot', 'balsam', 'banana', 'beef', 'bergamot', 'berry', 'bitter', 'black currant', 'brandy', 'bread', 'broth', 'burnt', 'butter', 'cabbage', 'camphor', 'caramel', 'cedar', 'celery', 'chamomile', 'cheese', 'chemical', 'cherry', 'chicken', 'chocolate', 'cinnamon', 'citrus', 'clove', 'cocoa', 'coconut', 'coffee', 'cognac', 'cooked', 'coumarin', 'cream', 'cucumber', 'dairy', 'earth', 'ester', 'ether', 'fat', 'fermented', 'fish', 'floral', 'flower', 'fruity', 'gardenia', 'garlic', 'gasoline', 'gassy', 'geranium', 'gourmand', 'grape', 'grapefruit', 'grass', 'green', 'hawthorn', 'hay', 'hazelnut', 'herbal', 'honey', 'horseradish', 'hyacinth', 'jam', 'jasmine', 'juice', 'ketonic', 'lactonic', 'lavender', 'leaf', 'lemon', 'licorice', 'lilac', 'lily', 'malt', 'marine', 'meat', 'medicinal', 'melon', 'menthol', 'metallic', 'milk', 'mimosa', 'mint', 'moss', 'muguet', 'mushroom', 'musk', 'musty', 'narcissus', 'neroli', 'onion', 'orange', 'orris', 'ozone', 'patchouli', 'peach', 'pear', 'peel', 'pepper', 'phenol', 'pine', 'pineapple', 'plastic', 'plum', 'popcorn', 'potato', 'pungent', 'raspberry', 'ripe', 'roasted', 'rooty', 'rose', 'rum', 'sandalwood', 'savory', 'sharp', 'smoked', 'solvent', 'sour', 'spicy', 'strawberry', 'sulfur', 'sweat', 'tea', 'terpene', 'tobacco', 'tomato', 'tropical', 'vanilla', 'vegetable', 'vetiver', 'violet', 'watery', 'wax', 'weedy', 'wine', 'woody']**- total of 146 descriptors**

Feature selection and Hyperparameter tuning

The features were filtered through an exhaustive feature selection procedure. 0 variance features were removed and then ANOVA F values were used to filter the features and the best feature for each of the 146 classes was selected. This was then narrowed down further through Recursive Feature Elimination, using a random forest with Permutation Feature Importance. The final set contained 23 features. The hyperparameter tuning of the models was done using Bayesian optimization through skopt [HEA].

Parameters for clustering odor descriptors in Data Driven Taxonomy
[n_clusters = 16, metric = 'euclidean', linkage ='ward']

Final features:

['BCUTc-1h', 'BCUTc-1l', 'BCUTZ-1h', 'BCUTv-1l', 'BCUTare-1l', 'RPCG', 'Xpc-4dv', 'Xp-1dv', 'Mare', 'ETA_shape_y', 'ETA_dAlpha_B', 'ETA_epsilon_4', 'SIC0', 'MIC1', 'SlogP_VSA5', 'EState_VSA5', 'VSA_EState6', 'VSA_EState7', 'VSA_EState8', 'nRot', 'GGI7', 'SRW04', 'SRW10']

Table S1. Search space and final parameters of the hyperparameter search for the XGBoost model using Bayesian Hyperparameter Optimization.

Hyperparameter	Search Space	Chosen Value
colsample_bynode	[0.1, 0.2, 0.3, 0.4, 0.5, 0.6, 0.7, 0.8, 0.9, 1.0]	0.9
learning_rate	[0.0001, 0.001, 0.01, 0.1, 0.2, 0.4, 0.6, 0.8]	0.4
max_depth	[3, 4, 5, 6, 7, 8, 9, 10, 11, 12]	3
min_child_weight	[1, 50, 100, 150, 200, 250]	1
n_estimators	[100, 200, 400, 600, 800, 1000, 2000, 4000, 5000, 10000]	2000
reg_lambda	[0.001, 0.01, 0.1, 1, 5, 10, 15, 20, 25]	5
subsample	[0.1, 0.2, 0.3, 0.4, 0.5, 0.6, 0.7, 0.8, 0.9, 1.0]	0.9
tree_method	['approx', 'hist']	‘hist’

Table S2. Search space and final parameters of the hyperparameter search for the Random Forest model using Bayesian Hyperparameter Optimization.

Hyperparameter	Search Space	Chosen Value
bootstrap	[True, False]	'False'
max_depth	[10, 20, 30, 40, 50, 60, 70, 80, 90, 100, None]	90
max_features	['log2', 'sqrt']	'log2'
min_samples_leaf	[1, 2, 3, 4]	1
min_samples_split	[2, 5, 10]	2
n_estimators	[50, 150, 200, 400, 600, 800, 1000, 1200, 1400, 1600, 1800, 2000]	1800

Table S3. Search space and final parameters of the hyperparameter search for the multi output Logistic Regression model using Bayesian Hyperparameter Optimization.

Hyperparameter	Search Space	Chosen Value
penalty	['l2']	'l2'
C	np.logspace(-4, 4, 20)	10000.0
solver	['lbfgs','newton-cg','liblinear','sag','saga']	'lbfgs'
max_iter	[100, 1000,2500, 5000]	2500

Table S4. Performance results using a **Logistic Regression model** after extensive feature selection and hyperparameter tuning.

Dataset	AUROC	F1 Score	Precision	Recall
**MMD**	0.527	0.079	0.170	0.059
**ET**	0.603	0.334	0.547	0.260
**DT**	0.609	0.319	0.528	0.262

Table S5. Performance results using a **Random Forest model** after extensive feature selection and hyperparameter tuning.

Dataset	AUROC	F1 Score	Precision	Recall
**MMD**	0.589	0.243	0.408	0.186
**ET**	0.676	0.488	0.601	0.421
**DT**	**0.693**	**0.511**	**0.623**	**0.444**

OpenPOM Model Performance

Application of OpenPOM using the available open-access implementation. For the sake of completeness, and to assess the performance of the expert taxonomy (ET) provided in the work with the current state-of-the-art model, we trained OpenPOM, an open source replication of the Principal Odor Map [LEE] with the MMD and both taxonomies (ET and DT). Random search cross-validation was used according to the provided example notebooks to find the optimal hyperparameters for MMD, DT and ET. The optimized hyperparameters for MMD, DT and ET are shown in Table S6-S8.

Table S6. Search space and final parameters of the hyperparameter search for the openPOM model using Random search CV for MMD.

Hyperparameter	Chosen Value
batch_size	128
loss_aggr_type	'sum'
node_out_feats	50
edge_hidden_feats	50
edge_out_feats	50
num_step_message_passing	1
mpnn_residual	True
message_aggregator_type	'mean'
mode	'classification'
number_atom_features	GraphConvConstants.ATOM_FDIM
number_bond_features	GraphConvConstants.BOND_FDIM
readout_type	'set2set'
num_step_set2set	5
num_layer_set2set	2
ffn_hidden_list	[392]
ffn_embeddings	256
ffn_activation	'relu'
ffn_dropout_p	0.5
ffn_dropout_at_input_no_act	False

weight_decay	1e-05
self_loop	False
optimizer_name	‘adam’

Table S7. Search space and final parameters of the hyperparameter search for the openPOM model using Random search CV for DT.

Hyperparameter	Chosen Value
batch_size	128
loss_aggr_type	‘sum’
node_out_feats	150
edge_hidden_feats	175
edge_out_feats	200
num_step_message_passing	2
mpnn_residual	False
message_aggregator_type	‘sum’
mode	‘classification’
number_atom_features	GraphConvConstants.ATOM_FDIM
number_bond_features	GraphConvConstants.BOND_FDIM
readout_type	‘set2set’
num_step_set2set	2
num_layer_set2set	1
ffn_hidden_list	[512, 512]
ffn_embeddings	256
ffn_activation	‘relu’
ffn_dropout_p	0.05
ffn_dropout_at_input_no_act	False
weight_decay	1e-05
self_loop	False
optimizer_name	‘adam’

Table S8. Search space and final parameters of the hyperparameter search for the openPOM model using Random search CV for ET.

Hyperparameter	Chosen Value
batch_size	128
loss_aggr_type	‘sum’
node_out_feats	200
edge_hidden_feats	75
edge_out_feats	200
num_step_message_passing	1
mpnn_residual	True
message_aggregator_type	‘sum’
mode	‘classification’
number_atom_features	GraphConvConstants.ATOM_FDIM
number_bond_features	GraphConvConstants.BOND_FDIM
readout_type	‘set2set’
num_step_set2set	2
num_layer_set2set	3
ffn_hidden_list	[512]
ffn_embeddings	256
ffn_activation	‘relu’
ffn_dropout_p	0.1625
ffn_dropout_at_input_no_act	False
weight_decay	1e-06
self_loop	True
optimizer_name	‘adam’

Label Pruning

To account for any performance gain that could result from the reduction in the output space, the MMD dataset was pruned off its labels to only include the top 16 labels. Since this would reduce the instances available for the machine learning task, the expert taxonomy was also randomly undersampled for a fair comparison. OpenPOM was used to compare the pruned dataset and the undersample ET imposed dataset. While the class distributions of the pruned and undersampled datasets were compared to ensure a similar class distribution, it would still not be an exact comparison. They are two different machine learning classification tasks, given that the metrics are based on the averaging of different classes as well as comparing a completely homogenous class as opposed to a semantically similar but heterogeneous class which adds some noise. However, this method will still give us a reasonable baseline to evaluate whether the taxonomy imposition provides a meaningful machine learning task or if the score is just in performance boost from the reduction of the output space.

Table S9. Performance metrics of the OpenPOM model for the pruned MMD as well as the undersampled ET imposed dataset.

Dataset	AUROC	F1	Precision	Recall
Pruned MMD	0.855	0.525	0.513	0.560
Undersampled ET	0.838	0.552	0.542	0.599

The results of the comparison between pruned MMD dataset and the undersampled ET dataset, shown in Table S9, show that the model trained on the undersampled ET gives a similar performance to that trained on the pruned MMD. This shows that the ET imposed dataset contains coherent enough classes to still perform similar to the pruned MMD despite having a more heterogenous, and hence noisier, class composition than the latter. Both of these tests show that the taxonomy imposed datasets constitute valid machine learning tasks and that there is a distinct signal and class separation in the new machine learning tasks.

Table S10. Classwise performance of the XGBoost model using the data-driven taxonomy. The 16 classes (A-P) as well as their scores across all metrics. The macro (unweighted) average of all the classes is provided in the last row.

Classes	AUROC	F1	Precision	Recall
A	0.659429	0.404762	0.485714	0.346939
B	0.674819	0.486275	0.529915	0.449275
C	0.726494	0.647120	0.674672	0.621730
D	0.654029	0.422330	0.446154	0.400922
E	0.702355	0.526971	0.569507	0.490348
F	0.664019	0.421569	0.505882	0.361345
G	0.717981	0.646245	0.670082	0.624046
H	0.708859	0.515406	0.557576	0.479167
I	0.756446	0.606335	0.697917	0.536000
J	0.720628	0.654369	0.693416	0.619485
K	0.699158	0.503704	0.596491	0.435897
L	0.708253	0.563107	0.610526	0.522523
M	0.641302	0.355140	0.431818	0.301587
N	0.885479	0.750000	0.709091	0.795918
O	0.696117	0.526733	0.588496	0.476703
P	0.558067	0.183908	0.320000	0.129032
**Average**	**0.698340**	**0.513373**	**0.567954**	**0.474432**

Table S11. Classwise performance of the XGBoost model using the expert driven taxonomy. The 16 classes as well as their scores across all metrics. The macro (unweighted) average of all the classes is provided in the last row.

Classes	**AUROC**	**F1**	**Precision**	**Recall**
Alcohol	0.697169	0.502283	0.526316	0.480349
Animal Body	0.668494	0.433566	0.553571	0.356322
Aquatic	0.587067	0.256410	0.454545	0.178571
Balsamic	0.685158	0.482385	0.542683	0.434146
Chemicals	0.644282	0.419913	0.451163	0.392713
Earthy	0.592802	0.284615	0.342593	0.243421
Flower	0.748530	0.650246	0.668354	0.633094
Fruity	0.738040	0.715421	0.748752	0.684932
Gourmand	0.674920	0.483940	0.548544	0.432950
Green	0.681113	0.631034	0.640981	0.621392
Herbal	0.668194	0.424581	0.506667	0.365385
Savory	0.763273	0.664935	0.684492	0.646465
Smoky	0.692512	0.472441	0.526316	0.428571
Spices	0.647363	0.397260	0.456693	0.351515
Sulfur	0.761012	0.588710	0.623932	0.557252
Woody	0.696945	0.528958	0.585470	0.482394
**Average**	**0.684180**	**0.496044**	**0.553817**	**0.455592**

Table S12. Odor descriptors falling under the 16 expert-driven classes.

Class	# descriptors	List of descriptors
**Alcohol**	10	['acid', 'sharp', 'pungent', 'brandy', 'cognac', 'ether', 'malt', 'rum', 'wine', 'alcohol']
**Animal Body**	4	['amber', 'musk', 'sweat', 'animal']
**Aquatic**	3	['fish', 'marine', 'watery']
**Balsamic**	2	['balsam', 'wax']
**Chemicals**	12	['ether', 'ozone', 'aldehyde', 'gasoline', 'ketonic', 'medicinal', 'metallic', 'phenol', 'plastic', 'solvent', 'terpene', 'chemical']
**Earthy**	5	['moss', 'mushroom', 'musty', 'earth', 'rooty']
**Flower**	15	['geranium', 'lavender', 'mimosa', 'narcissus', 'orris', 'rose', 'violet', 'gardenia', 'hyacinth', 'jasmine', 'lilac', 'lily', 'muguet', 'flower', 'floral']
**Fruity**	28	['berry', 'cherry', 'black currant', 'raspberry', 'strawberry', 'bergamot', 'citrus', 'grapefruit', 'lemon', 'neroli', 'orange', 'peel', 'apple', 'apricot', 'banana', 'coconut', 'ester', 'grape', 'hawthorn', 'juice', 'melon', 'peach', 'pear', 'pineapple', 'plum', ['ripe','tropical', 'fruity']
**Gourmand**	13	'almond', 'roasted', 'bitter', 'caramel', 'cocoa', 'cream', 'hazelnut', 'honey', 'jam', 'popcorn', 'vanilla', 'chocolate', 'gourmand']
**Green**	9	['celery', 'cucumber', 'grass', 'green', 'leaf', 'herbal', 'weedy', 'coumarin', 'hay']
**Herbal**	5	['menthol', 'mint', 'chamomile', 'tea', 'vetiver']
**Savory**	23	['garlic', 'onion', 'cooked', 'cabbage', 'roasted', 'bread', 'butter', 'cheese', 'cream', 'milk', 'lactonic', 'sour', 'fat', 'peel', 'beef', 'chicken', 'meat', 'broth', 'vegetable', 'potato', 'tomato', 'dairy', 'savory']
**Smoky**	5	['burnt', 'smoked', 'coffee', 'roasted', 'tobacco']
**Spices**	8	['anisic', 'cinnamon', 'clove', 'horseradish', 'licorice', 'pepper', 'anise', 'spicy']
**Sulfur**	3	['fermented', 'gassy', 'sulfur']
**Woody**	9	['camphor', 'cognac', 'patchouli', 'rooty', 'sandalwood', 'woody', 'lactonic', 'cedar', 'pine']

As described in Section 2.2.1 of the ms. for the expert taxonomy on the ODEUROPA website, the source-based descriptors were divided in 16 different classes 'scent families') and 31 Subclasses, e.g., the “Alcohol” class contains the sub-classes “Acid” and “Alcohol”, which each contains their own odor descriptors. The classes (**bold**) and sub-classes (*italic*) are provided below, the hierarchical expert taxonomy is provided on the ODEUROPA website.

1. **Alcohol**
    - *1.1. Acid*
    - 1.2. *Alcohol*
2. **Animal**
    - *2.1. Animal*
    - *2.2. Body*
3. **Aquatic**
    - *3.1. Fish*
    - *3.2. Sea*
4. **Balsamic**
5. **Chemical**
    - *5.1. Ether*
    - *5.2. Other*
6. **Earthy**
    - *6.1. White flowers*
    - *6.2. Other*
7. **Flower**
    - *7.1. Berry*
    - *7.2. Citrus*
    - *7.3. Other*
8. **Fruity**
    - *8.1. Ether*
    - *8.2. Other*
9. **Gourmand**
10. **Green**
    - *10.1. Grass*
    - *10.2. Hay*
11. **Herbal**
    - *11.1. Methol*
    - *11.2. Other*
12. **Savory**
    - *12.1. Allium*
    - *12.2. Brassica*
    - *12.3. Bread*
    - *12.4. Dairy*
    - *12.5. Fat*
    - *12.6. Meat*
    - *12.7. Umami*
    - *12.8. Other*
13. **Smoky**
14. **Spices**
15. **Sulfur**
    - *15.1. Decay*
    - *15.2. Excrement*
    - *15.3. Sulfur*
16. **Woody**
    - *16.1. Ether*
    - *16.2. Other*

Table S13. Descriptors falling under the 16 data driven classes.

Class	# descriptors	List of descriptors
**A**	12	['almond', 'anise', 'anisic', 'bitter', 'cherry', 'hawthorn', 'hyacinth', 'licorice', 'lilac','mimosa', 'narcissus', 'plastic']
**B**	14	['bread', 'burnt', 'caramel', 'chocolate', 'cocoa', 'coffee', 'earth', 'gourmand', 'hazelnut', 'malt', 'mushroom', 'musty', 'popcorn', 'potato']
**C**	22	['bergamot', 'black currant', 'celery', 'chamomile', 'floral', 'flower', 'gardenia', 'geranium', 'grape', 'grapefruit', 'honey', 'jasmine', 'lavender', 'lily', 'muguet', 'neroli','orris', 'plum', 'rose', 'tea', 'tobacco', 'violet']
**D**	13	['cabbage', 'chemical', 'fish', 'gasoline', 'gassy', 'horseradish', 'ketonic', 'metallic', 'pepper', 'pungent', 'sharp', 'tomato', 'vegetable']
**E**	8	['balsam', 'cinnamon', 'clove', 'medicinal', 'phenol', 'smoked', 'spicy', 'vanilla']
**F**	8	['butter', 'coconut', 'coumarin', 'cream', 'dairy', 'hay', 'lactonic', 'milk']
**G**	8	['cucumber', 'fat', 'grass', 'green', 'leaf', 'melon', 'wax', 'weedy']
**H**	8	['alcohol', 'brandy', 'cognac', 'ether', 'fermented', 'rum', 'solvent', 'wine']
**I**	7	['beef', 'broth', 'chicken', 'cooked', 'meat', 'roasted', 'savory']
**J**	11	['apple', 'apricot', 'banana', 'ester', 'fruity', 'juice', 'peach', 'pear', 'pineapple', 'ripe', 'tropical']
**K**	8	['aldehyde', 'citrus', 'lemon', 'marine', 'orange', 'ozone', 'peel', 'watery']
**L**	6	['camphor', 'herbal', 'menthol', 'mint', 'pine', 'terpene']
**M**	4	['acid', 'cheese', 'sour', 'sweat']
**N**	3	['garlic', 'onion', 'sulfur']
**O**	10	['amber', 'animal', 'cedar', 'moss', 'musk', 'patchouli', 'rooty', 'sandalwood', 'vetiver', 'woody']
**P**	4	['berry', 'jam', 'raspberry', 'strawberry']

The prompt used to generate the class names with ChatGPT (GPT-4o) is the following:
"I am working on a taxonomy (or ontology) to describe the conceptual hierarchy between odor descriptors for molecules. This will be leveraged to improve machine learning models for structure-odor predictions. I have groups of several descriptors, e.g., "rose", "meat", "blackcurrant" etc and I want to give a name to each group, an umbrella term under which different descriptors can fall. The descriptors' groups are the following: [...]
I need only a single word to best describe each group, with vocabulary that is mainly associated with perfumery and smell experts. Please go in the right order that I provide."

See Section 3.2 of the ms. and Table 3 for the list of class names.

Methodology and Workflow.

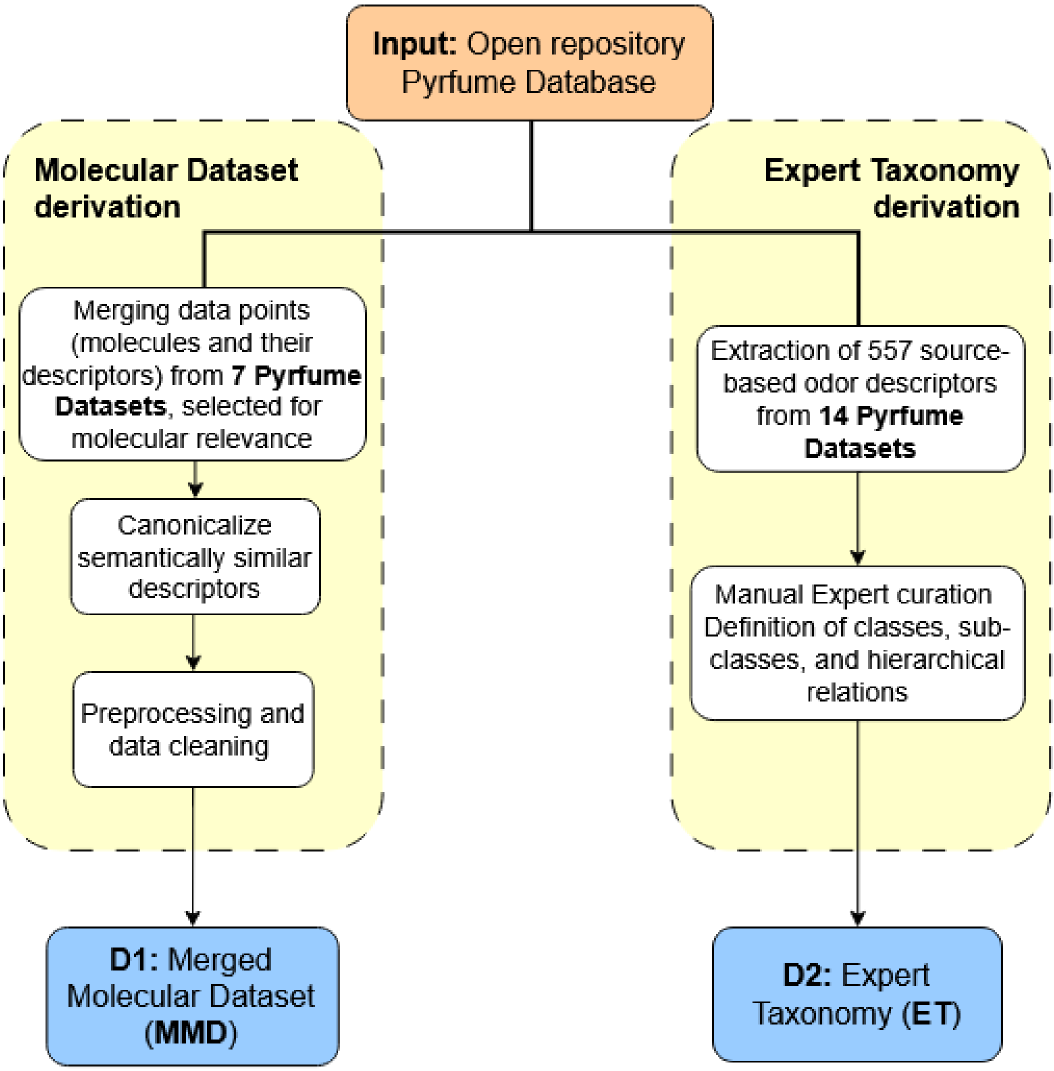

Figure S1. Schematic Workflow derivation of the main deliverables provided in this work. **D1:** the merged molecular data set (MMD) that contains 6711 molecules and 146 odor descriptors; **D2:** Curated Expert taxonomy providing hierarchical relations between 557 odor descriptors. To derive the MMD, 7 useful molecular datasets were merged from the pyrfume database that contained useful molecular information, while 14 different data sets were used to compile the ET.

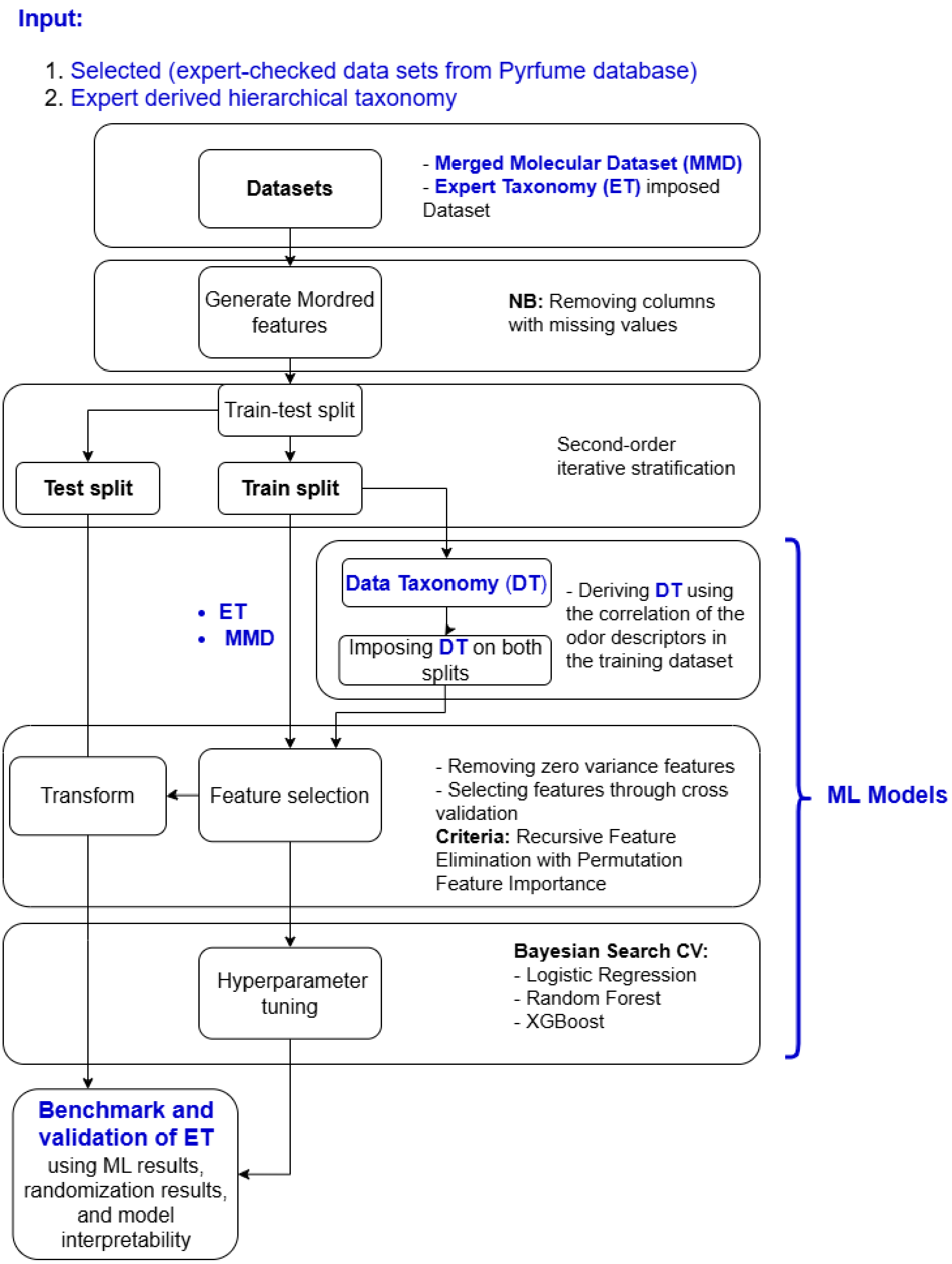

Figure S2. Schematic workflow of the machine learning pipeline used to assess the usefulness of an expert-derived odor taxonomy (ET) by comparing the usefulness of the ET with a data-driven taxonomy (DT) across multiple models, feature-selection strategies, and interpretability analyses (see methods section of the ms. for additional details).

Randomization convergence. The total number of possible randomized taxonomies that can be generated using the odor descriptors in their respective parent term is 146 factorial (146!). However, to get an overview of the score distribution of each metric it is not necessary to use all the 146! possible combinations. According to the central limit theorem [KWA], the sample distribution converges to the population distribution with increasing sample size. It is therefore possible to approximate the population mean with a lower number of samples. To verify if the running mean and running standard deviation for all metrics have converged, we use 1000 randomized taxonomies. The randomization convergence plots of the ET (1000), DT (1000) and the combined taxonomies (2000) are shown below.

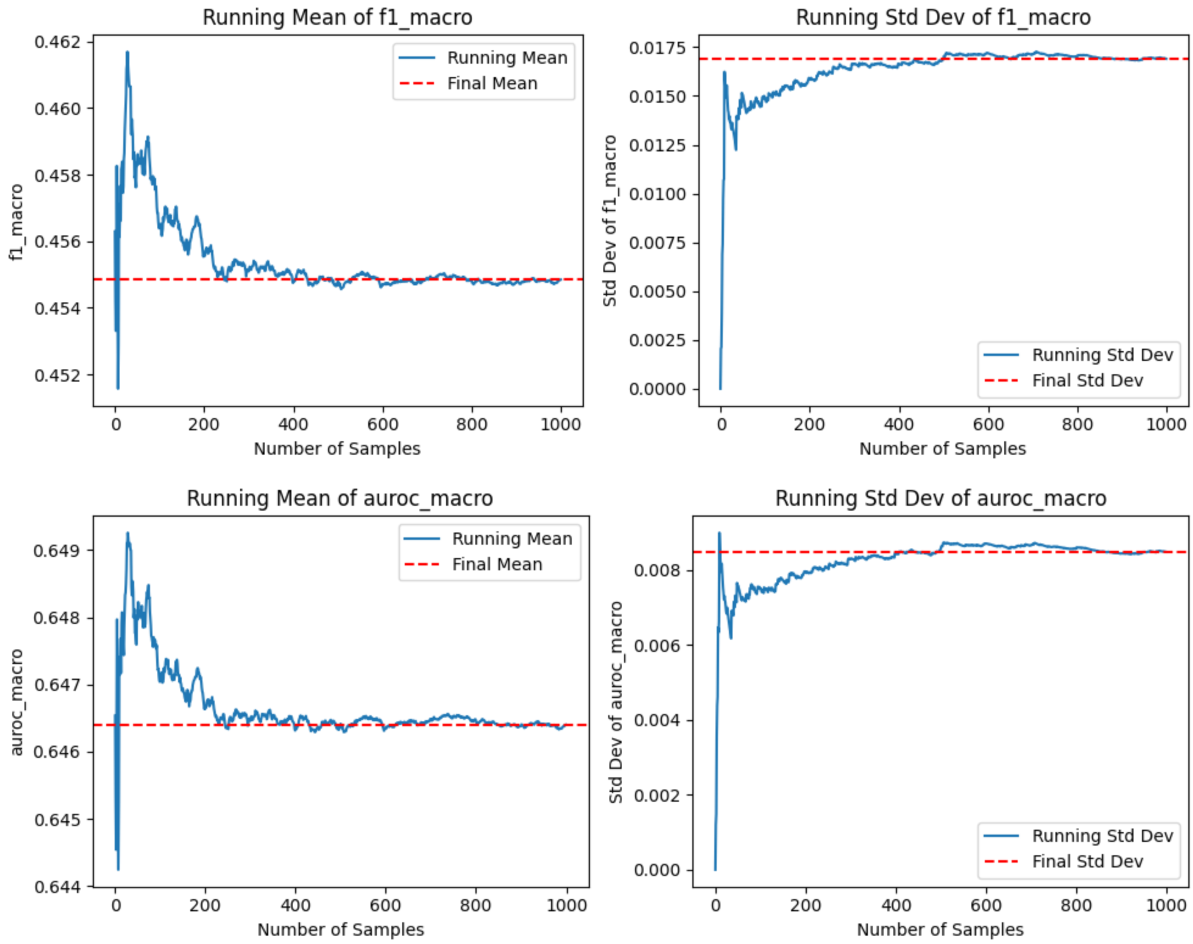

Figure S3-1. Development of the mean and standard deviation with the addition of each new metric score for 1000 randomizations on the expert-driven taxonomy. Macro metrics F1 and AUROC over number of samples (randomized taxonomies). Note that all metrics converge, showing that the score metrics can be taken as a close representative of the randomizations, or the gain in score metrics due to reducing the number of classes.

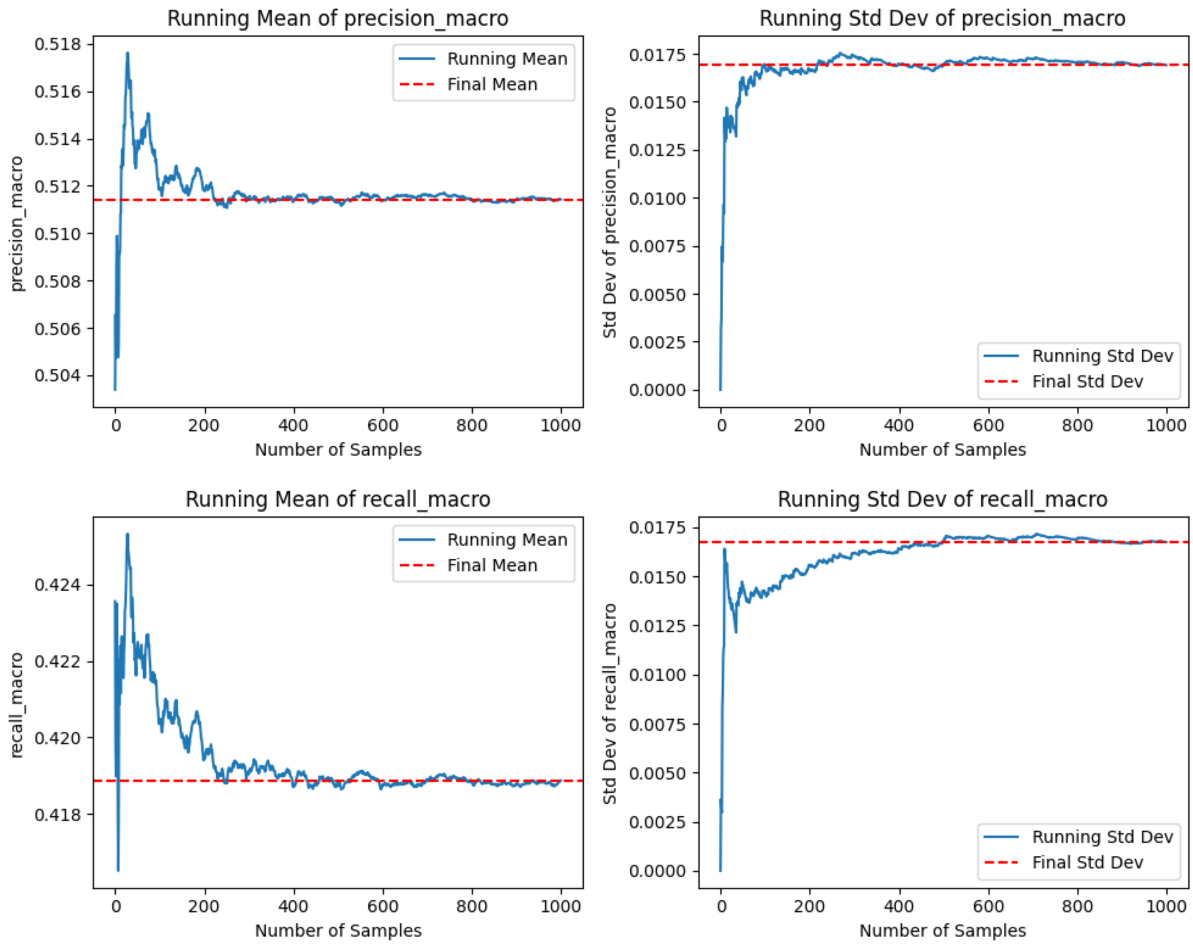

Figure S3-2. Macro metrics precision and recall (randomization expert-driven taxonomy). See Caption Figure S1-1 for detailed description.

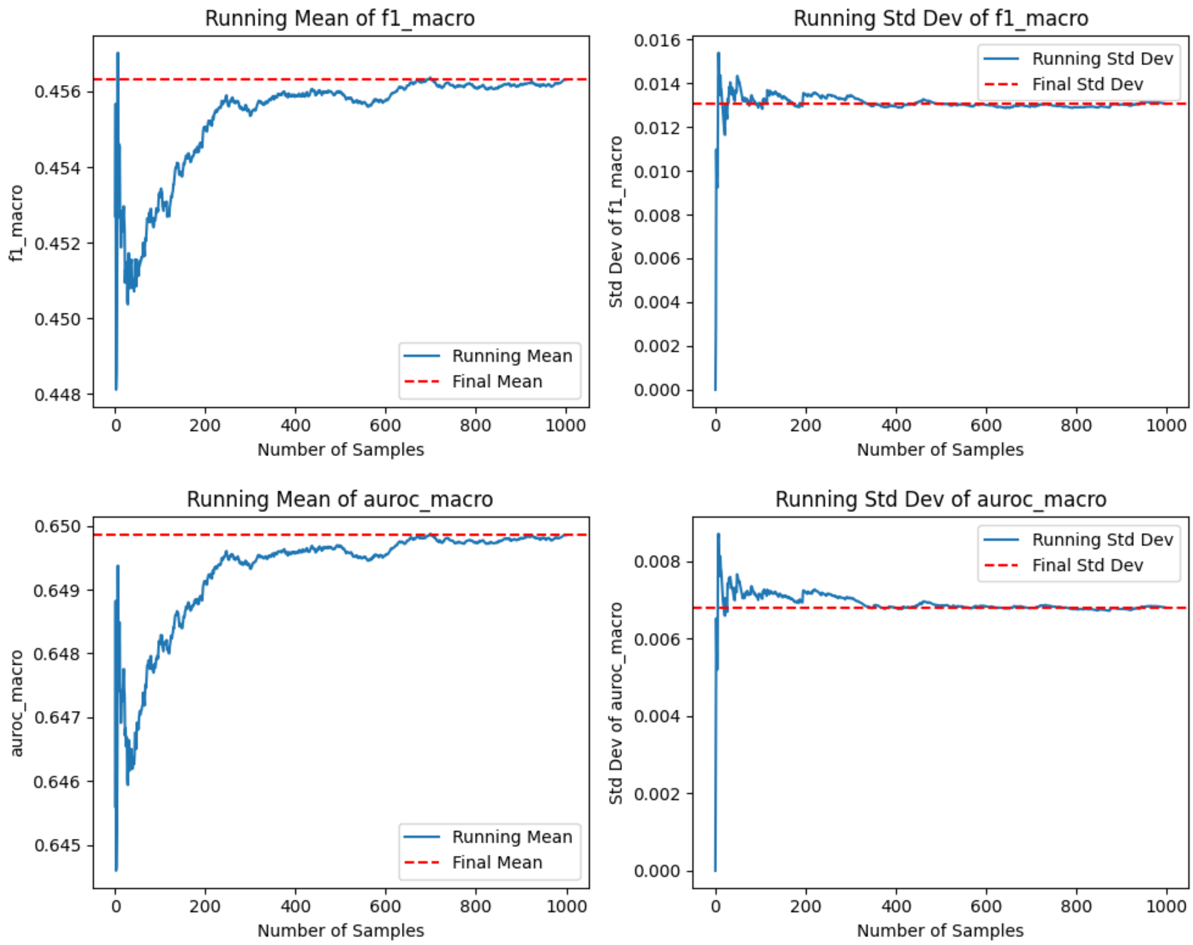

Figure S4-1. Development of the mean and standard deviation with the addition of each new metric score for 1000 randomizations on the data driven taxonomy. Macro metrics F1 and AUROC over number of samples (randomized taxonomies). Note that all metrics converge, showing that the score metrics can be taken as a close representative of the randomizations, or the gain in score metrics due to reducing the number of classes.

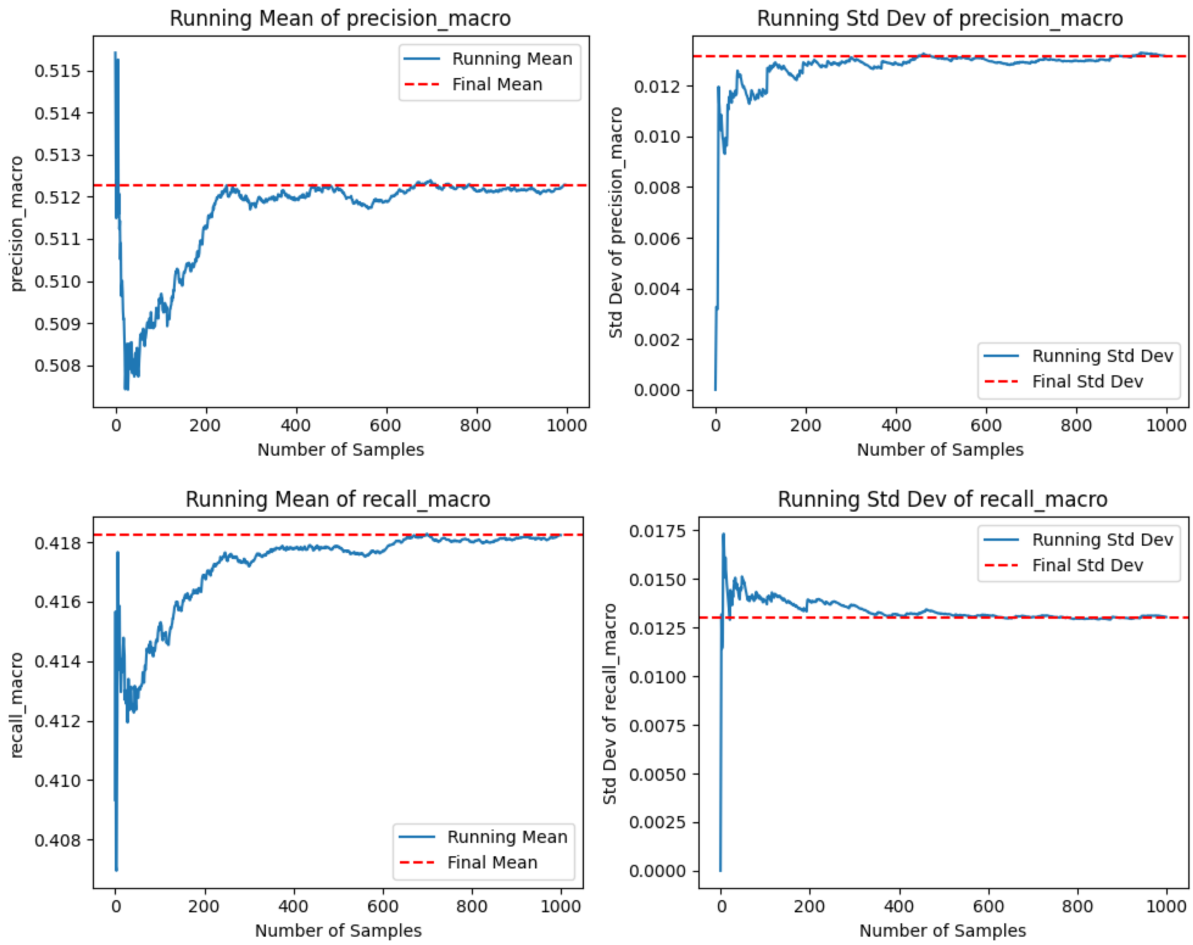

Figure S4-2. Macro metrics precision and recall (randomization data-driven taxonomy). See Caption Figure S2-1 for detailed description.

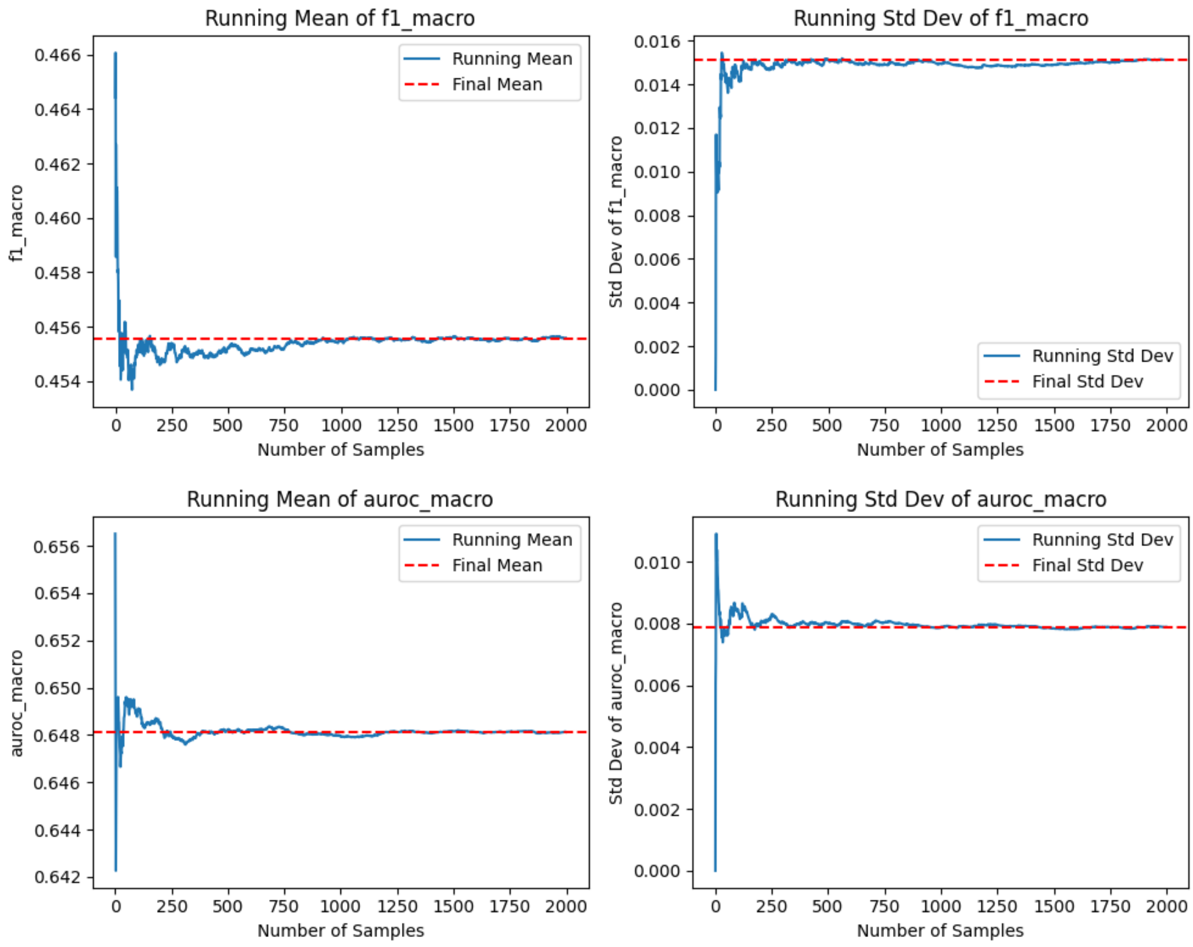

Figure S5-1. Mean and standard deviation over 2000 randomizations taxonomies combining the macro metrics F1 and AUROC for both the expert and the data-driven taxonomy (see Figures S1 and S2, respectively). This shows that the joint distribution converges and can be used to represent the performance gain from reducing class count.

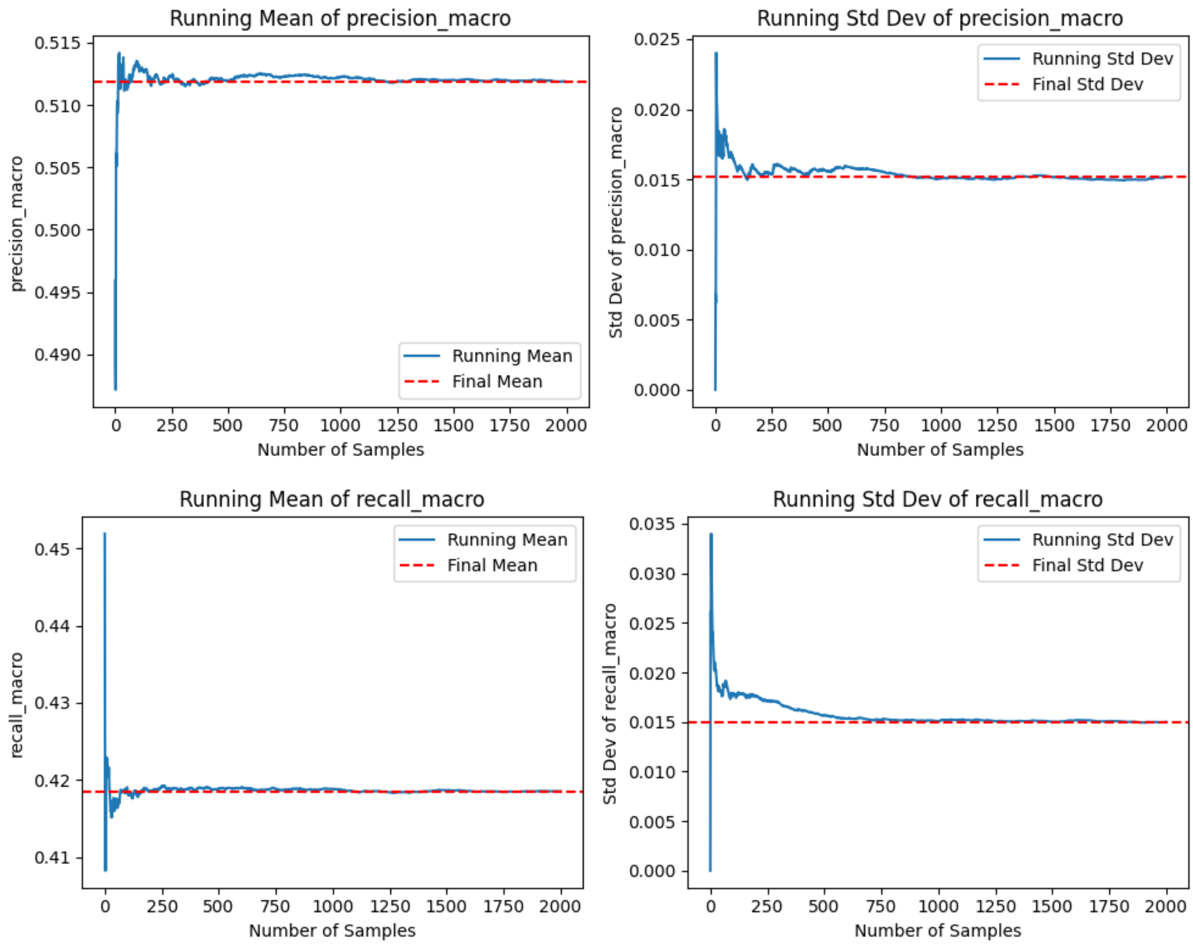

Figure S5-2. Mean and standard deviation over 2000 randomizations taxonomies combining the macro metrics precision and recall for both the expert and the data-driven taxonomy (see Figures S1 and S13, respectively). See Caption Figure S3-1 for detailed description.

Data Exploration

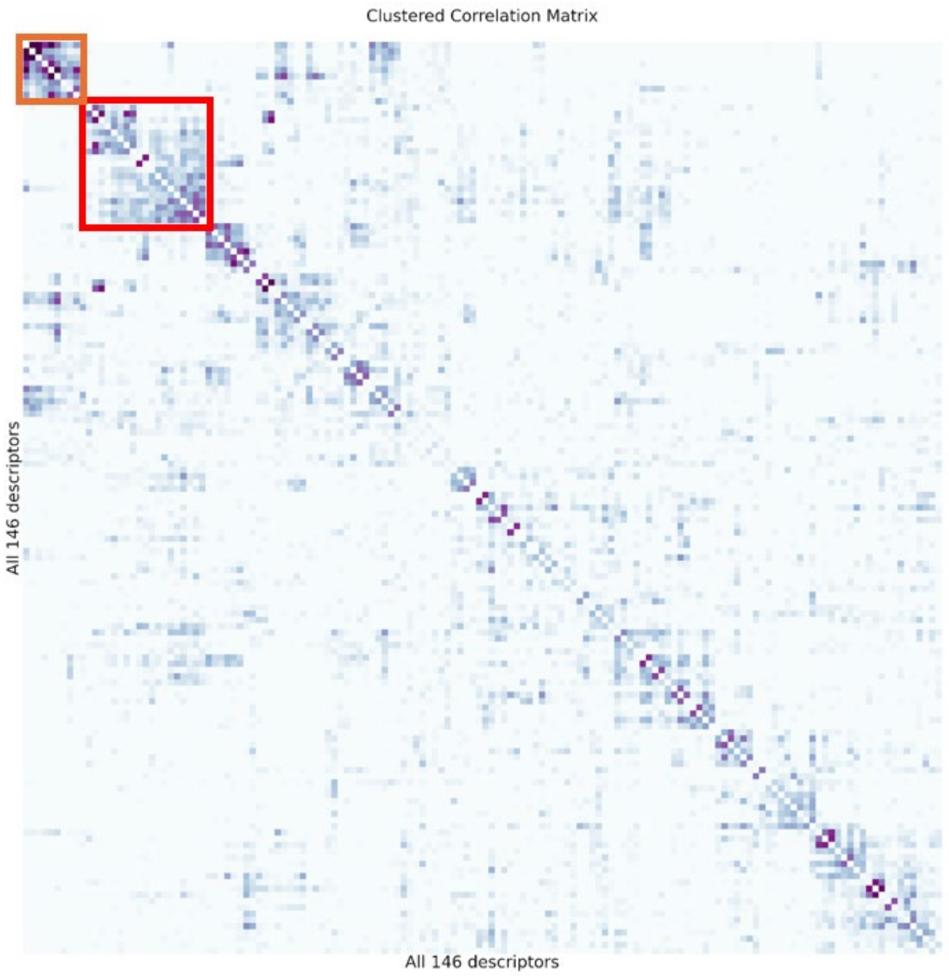

Figure S6. Clustered correlation plot of all 146 descriptors using the co-occurrence from the training dataset. The co-occurrence of similar odor descriptors allows for a more perceptually meaningful clustering. The two clusters highlighted in orange and red correspond to the sulfur/savory and the alcohol/fruity clusters, respectively. For a zoom in on the alcohol and the fruity clusters see manuscript (Figure 4).

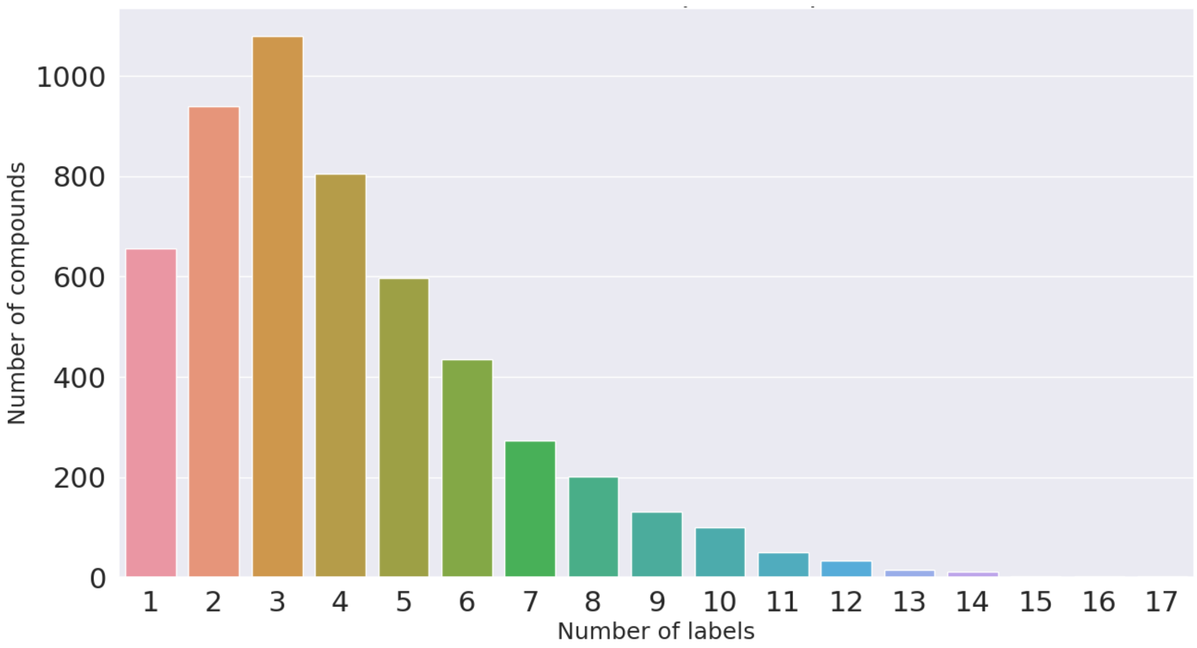

Figure S7. Overview of chemical compounds and the number of corresponding labels or descriptors provided in the full training dataset (5331 molecules - see ms. for GitHub repository). The majority of compounds have between 1 and 5 labels. 32 compounds have 13 labels or more with a maximum number of 17 descriptors.

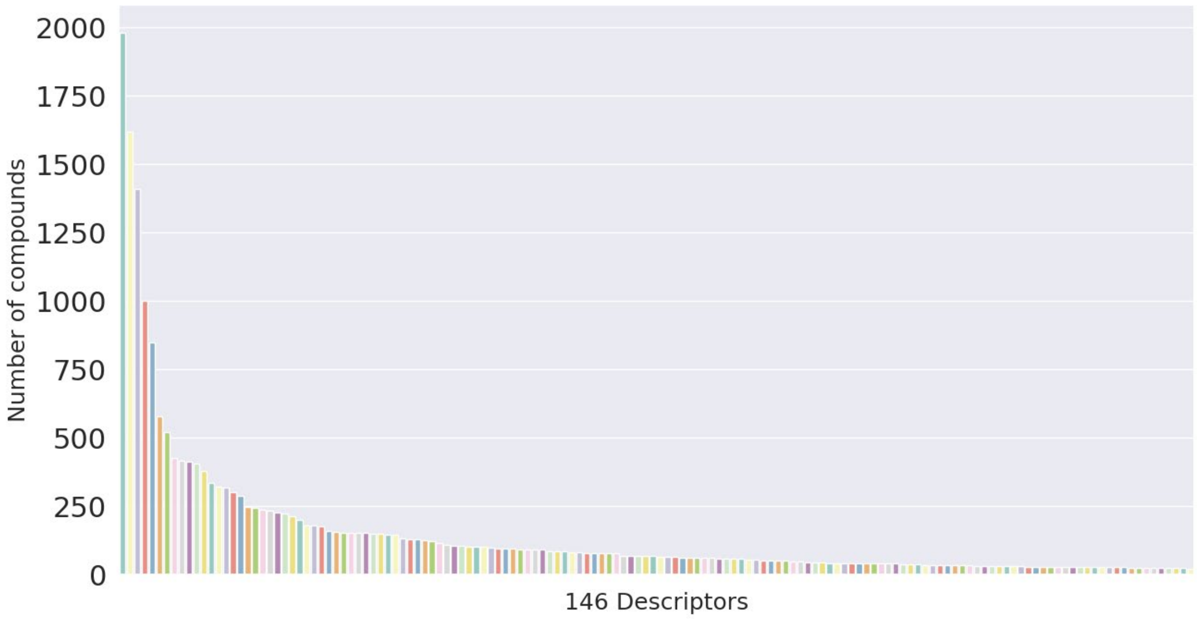

Figure S8. Number of compounds in each of the different classes, i.e., provided smell descriptors, for a total of 146 descriptors. It should be noted that the number of instances throughout the classes are highly imbalanced.

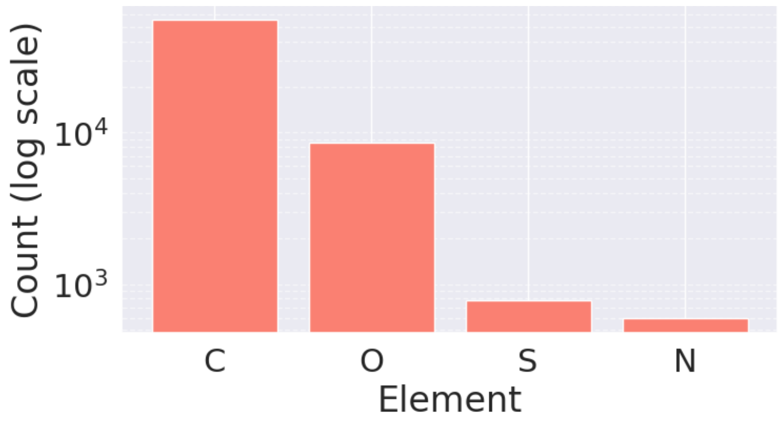

Figure S9. Total sum of the number of carbon (C), oxygen (O), sulphur (S), and nitrogen (N) atoms within the all-descriptor training dataset (5331 molecules).

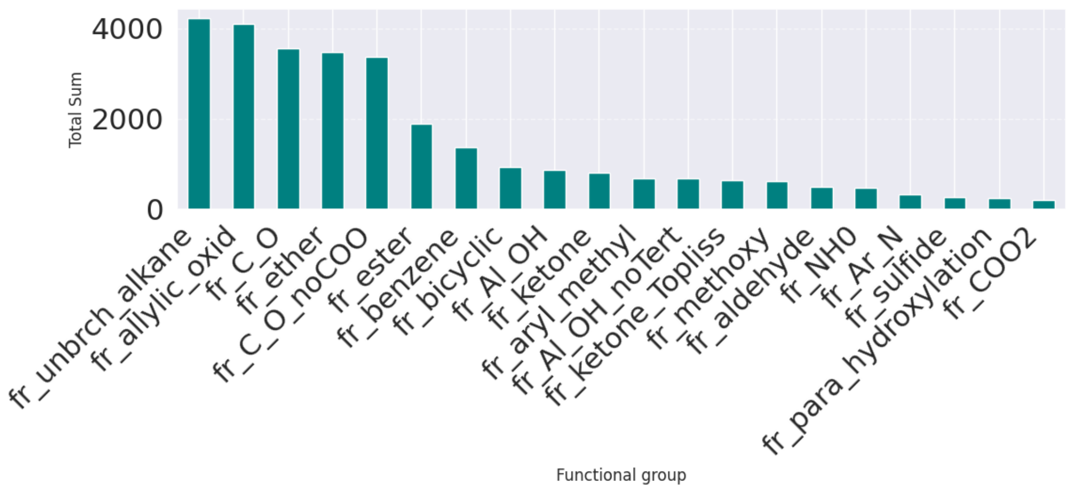

Figure S10. Number of different functional groups present in the training data set (5331 compounds - 80% of the full dataset with 6711 molecular compounds).

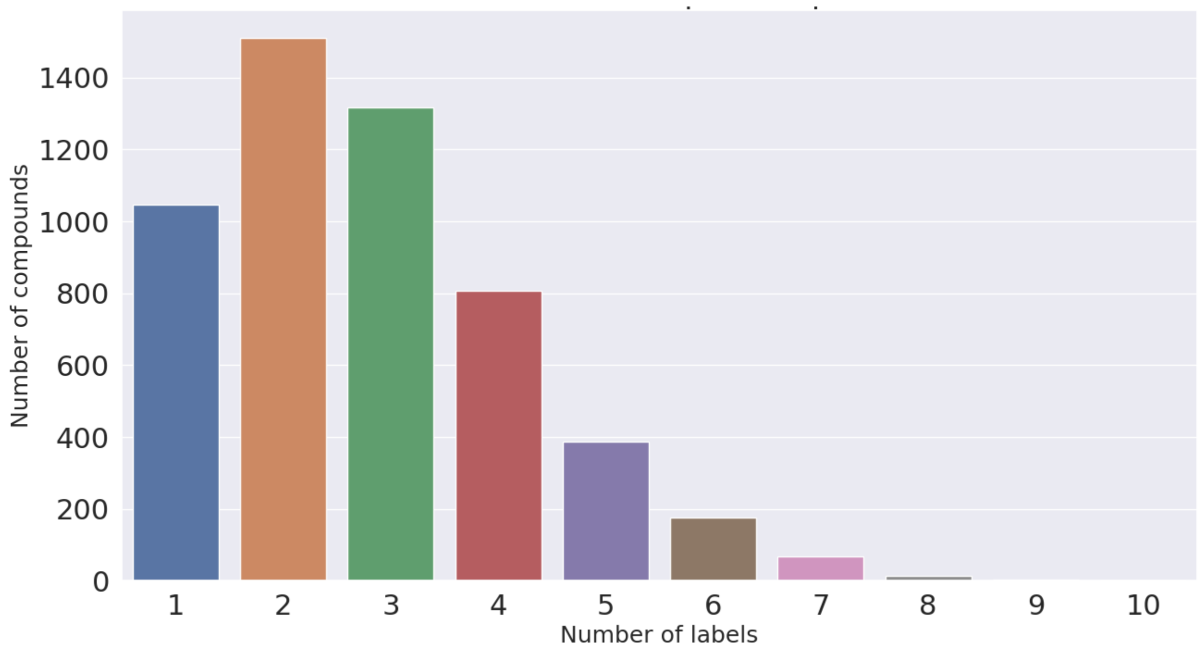

Figure S11. Overview of chemical compounds and the number of corresponding labels or descriptors provided in the data set imposed using the data-driven taxonomy. The majority of compounds have between 1 and 4 labels. See Figure S13 for the distribution of the labels obtained using the expert taxonomy.

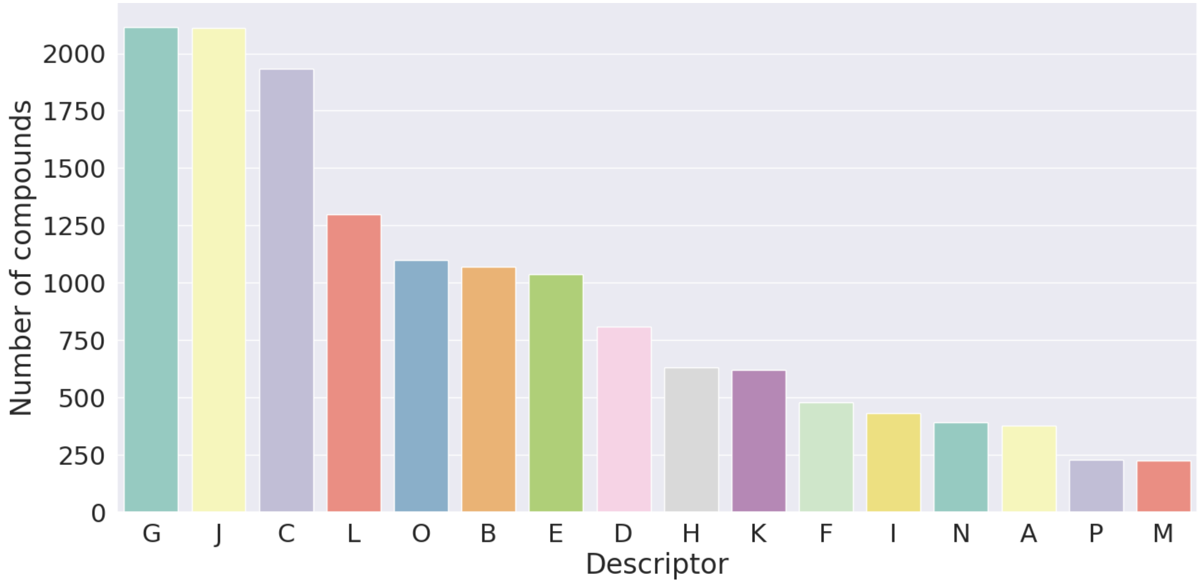

Figure S12. Number of compounds in the 16 different classes using the data driven taxonomy. It should be noted that the classes are still imbalanced. See Figure S14 for the distribution of compounds in the 16 expert-driven classes.

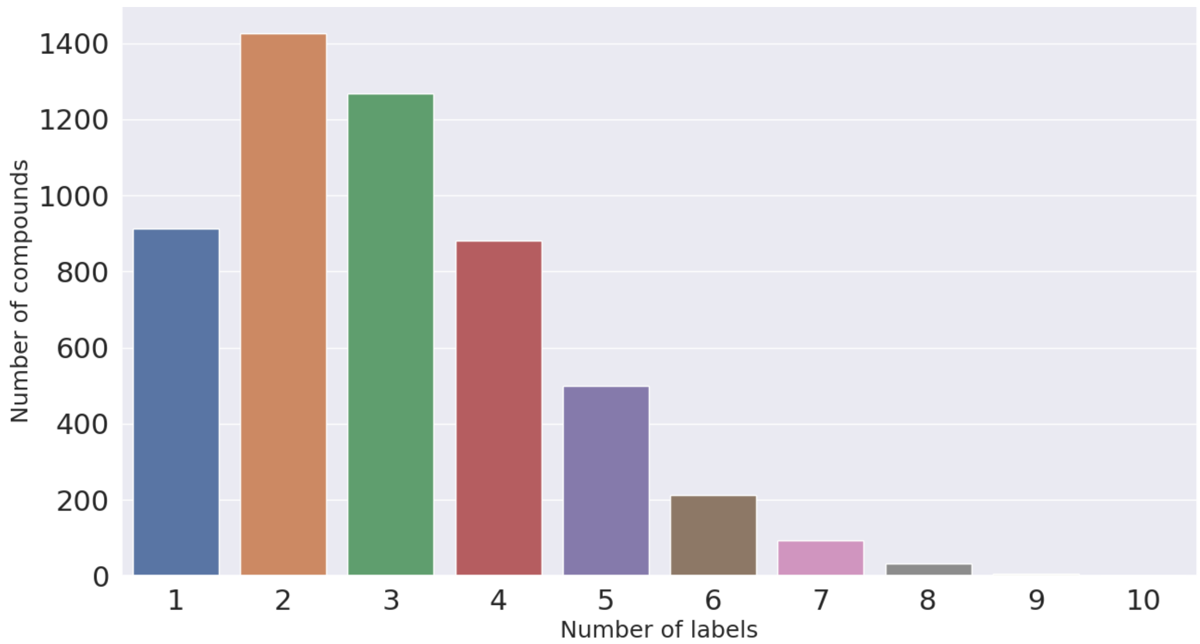

Figure S13. Overview of chemical compounds and the number of corresponding labels or descriptors provided for the dataset imposed with the expert-driven taxonomy. The majority of compounds have between 1 and 4 labels similar to that of the data-driven taxonomy. Likewise, the upper bound of the possible number of labels has decreased from 15-17 in the dataset with all the descriptors to 8-10.

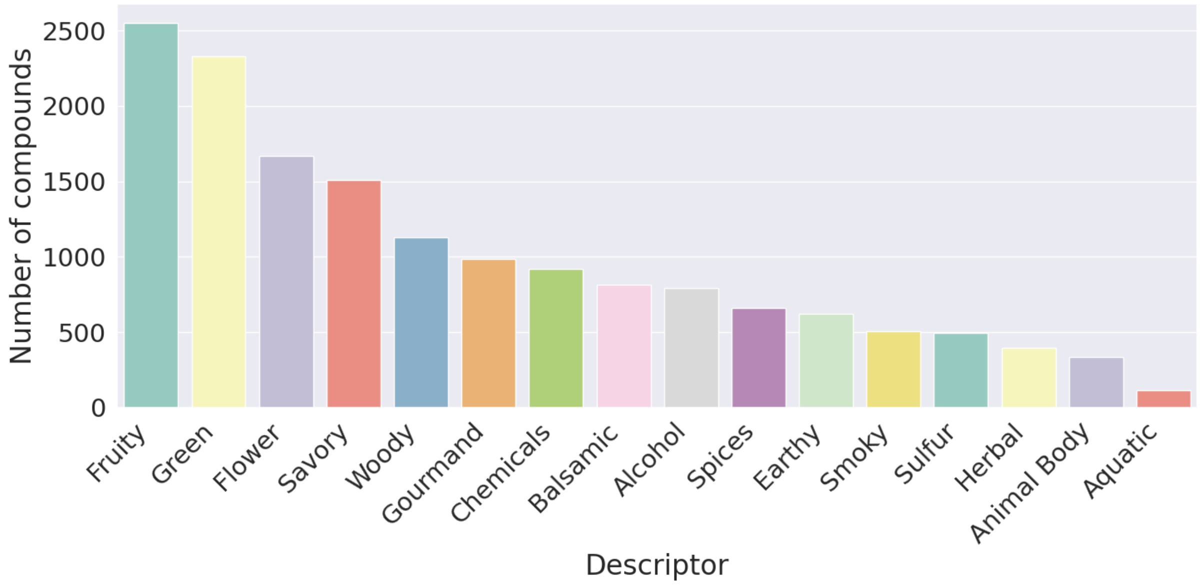

Figure S14. Number of compounds in each of the different classes, i.e., provided smell descriptors, for a total of 16 parent term descriptors of the expert taxonomy. It should be noted that the classes are still imbalanced.

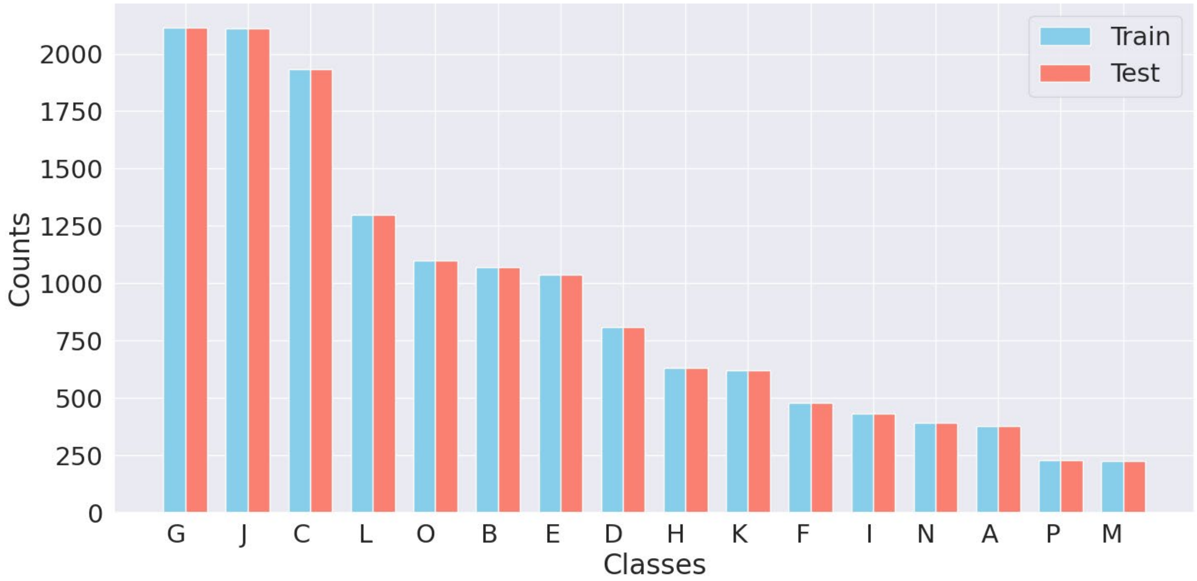

Figure S15. Number of compounds in each of the different classes using the DT across the train and test splits.

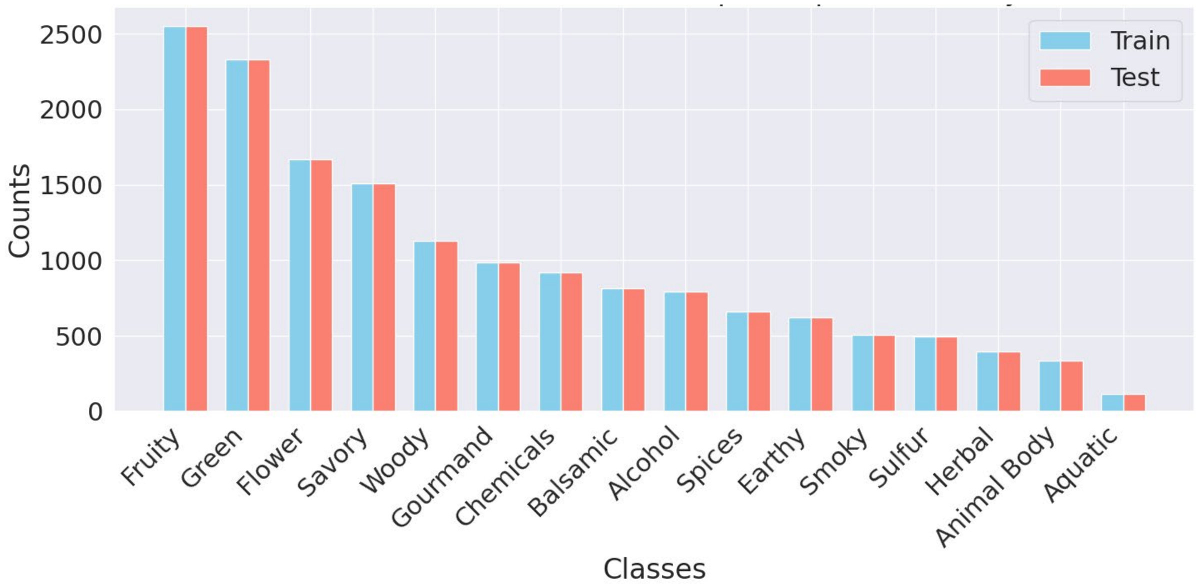

Figure S16. Number of compounds in each of the different classes using the expert taxonomy across both train and test splits.

ERROR ANALYSIS AND FEATURE IMPORTANCE

To investigate the most relevant features that the machine learning model uses for its predictions, the model's feature importance is looked at. However, since the feature importance of the model is based on how the model fits the training dataset, it's not as reliable. Permutation feature importance (PFI) proves a more reliable technique, where permutations are added to the features of the test data to rank features based on how much a given score metric drops upon permuting a given feature. The limitation of the PFI is that it looks at the drop of overall macro scores for evaluating feature importance and there is no class specificity. Therefore, SHAP Value Analysis are used in a second step (see further below).

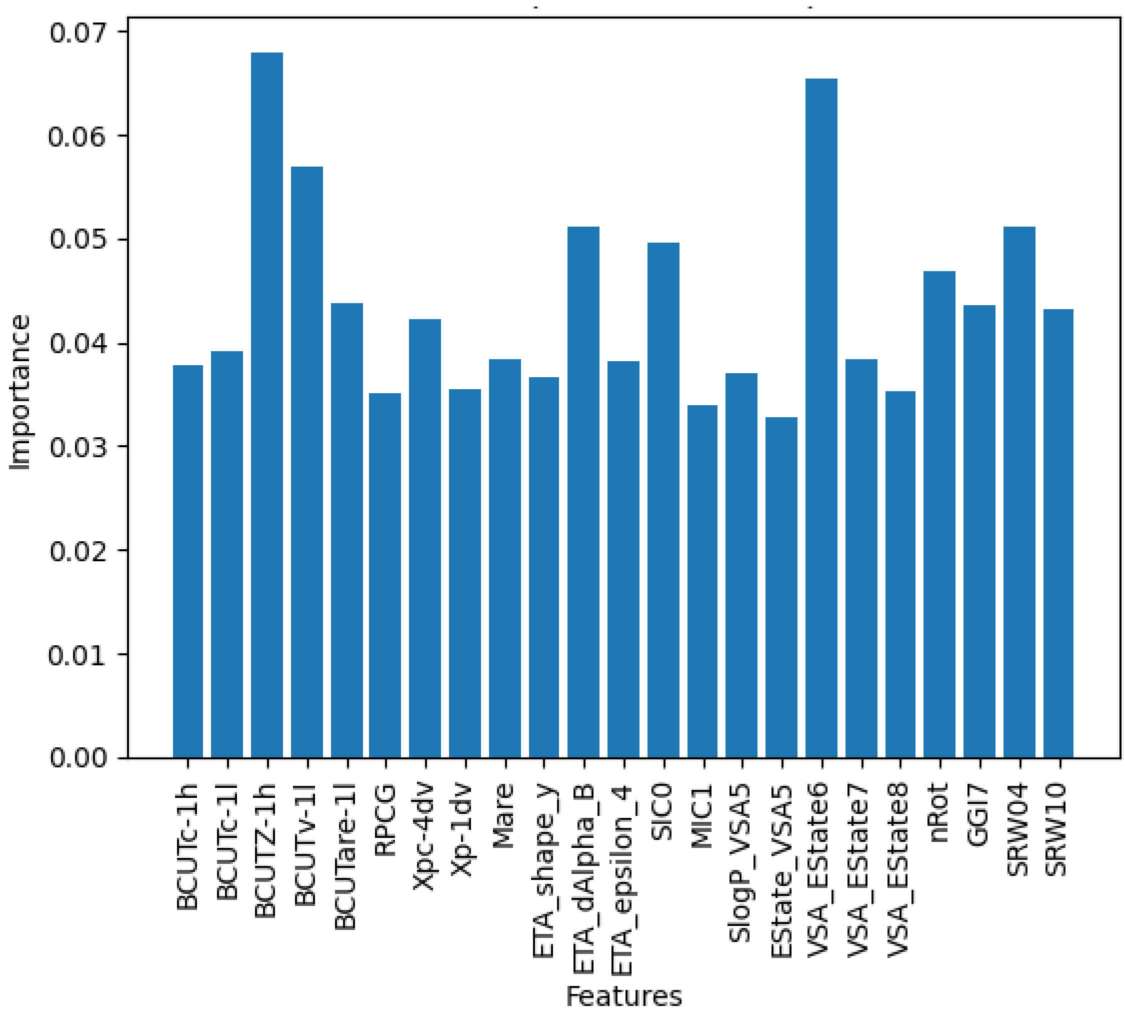

Figure S17. Feature importance of the XGBoost model for the data-driven Taxonomy. The plot shows that the features BCUTZ-1h, BCUTv-1l and VSA-Estate6 are relevant for the model to complete the classification task. Note that this is based on the training data alone.

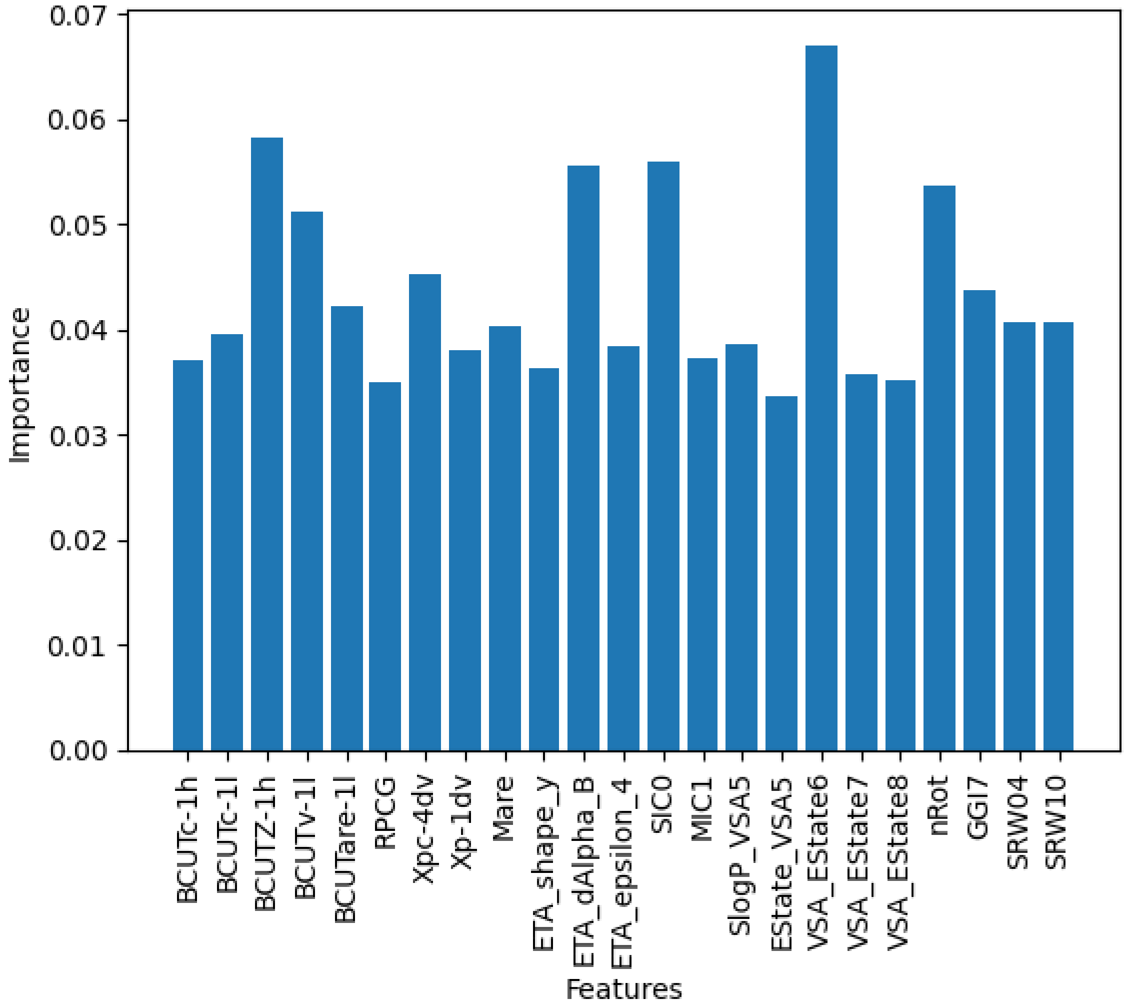

Figure S18. Feature importance of the XGBoost model for the expert-driven Taxonomy. The plot shows that the features BCUTZ-1h, SIC0 and VSA-Estate6 are relevant for the model to complete the classification task. Note that this is based on the training data alone.

SHAP Value Analysis. The SHapley Additive exPlanations (SHAP) analysis employs a game-theoretic approach to assign SHAP values, which represent the contribution or importance of each feature to a machine learning model's output [LUN]. The resulting values can be used to interpret the model predictions. We provide the SHAP summary plots for the different classes in both the computational and expert taxonomies. The features are listed on the y-axis, while the x-axis indicates the magnitude and direction of their contribution to the performance output of the XGBoost classifier. Each point in the plots represents an individual data instance, with color indicating the actual feature value (red for high, blue for low). Features are ranked based on importance, with those at the top contributing most significantly. The horizontal spread of points reflects the extent of a feature's influence across the dataset, with wider distributions imply greater overall impact on model predictions.

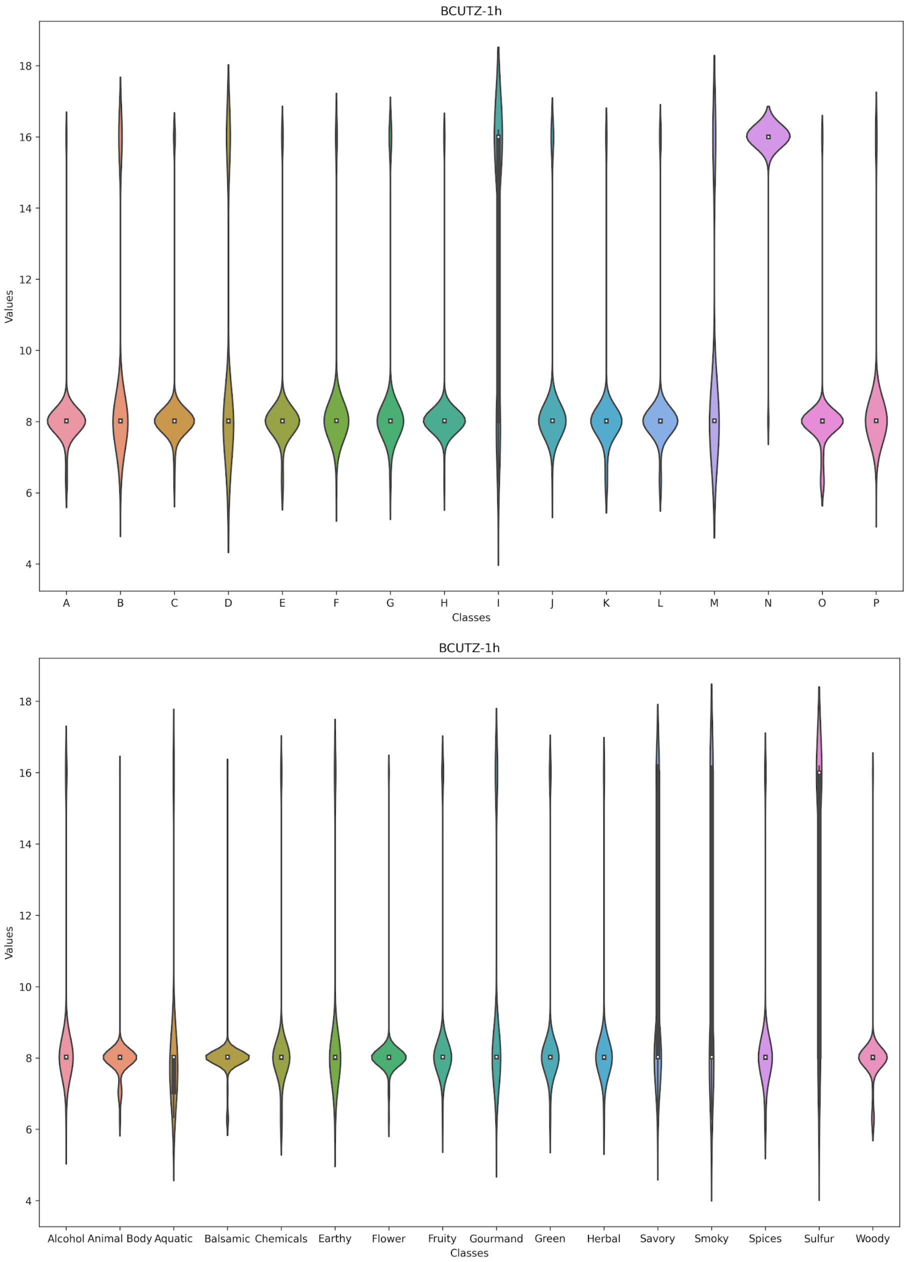

Figure S19. The feature ‘BCUTZ-1h’ across both taxonomies, data and expert-driven (upper and lower part, respectively). This is the most defining feature for the ‘Sulfur’ class across both taxonomies, corresponding to the atomic number of the atoms in the molecule samples, 16 in the case of sulfur atoms. For the DT, we can see that a lot of data points cluster around 16 for the ‘N’ class which conceptually corresponds to the Sulfur class, as well as for the ‘I’ class, which corresponds to the “Savory” class of the expert taxonomy.

The remaining pages consist of classwise SHAP plots for all classes across both taxonomies which individually show feature contributions for the model's decision making.

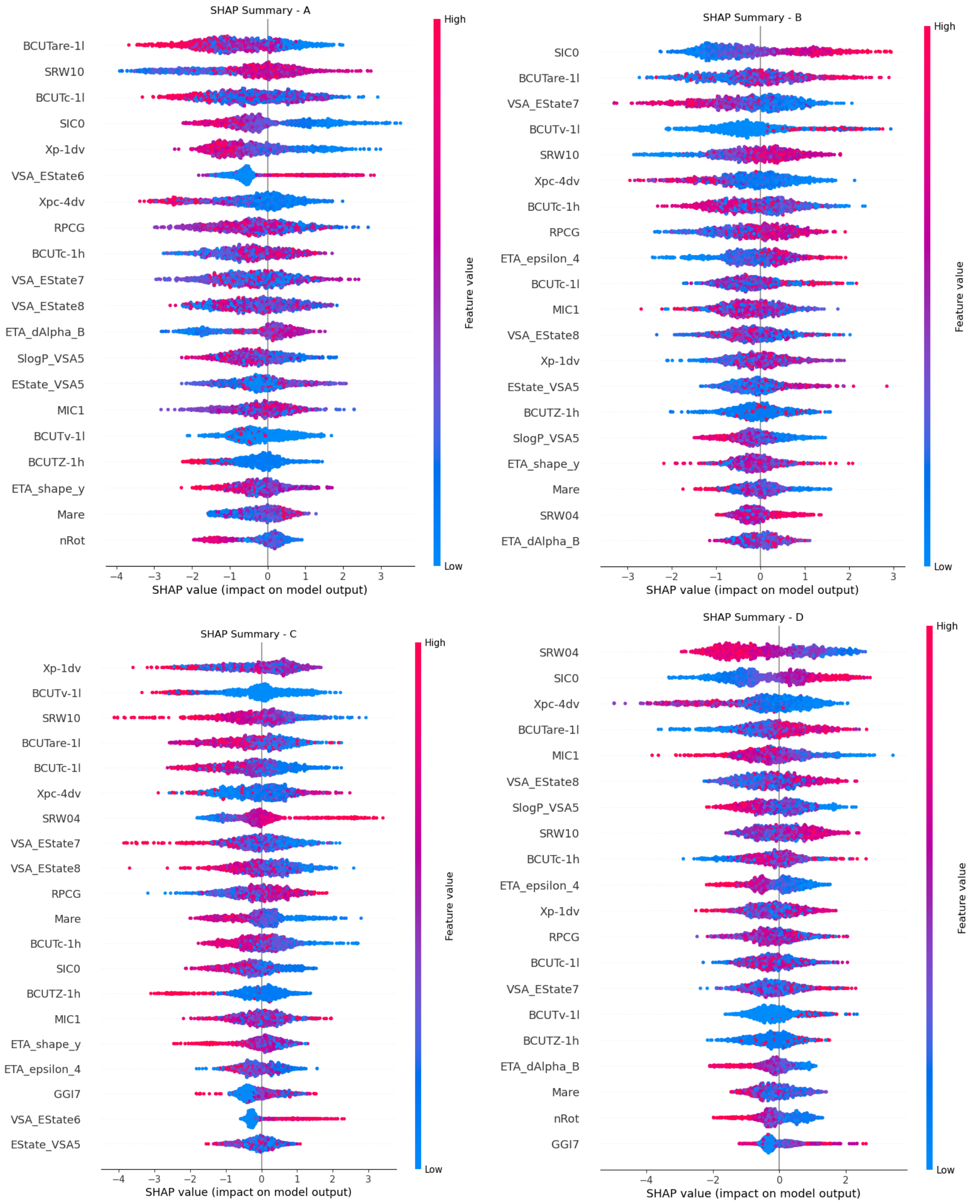

Figure S20-1: SHAP analysis of the XGBoost classifier on the DT for all 16 classes. See also manuscript for the available code on the GitHub repository.

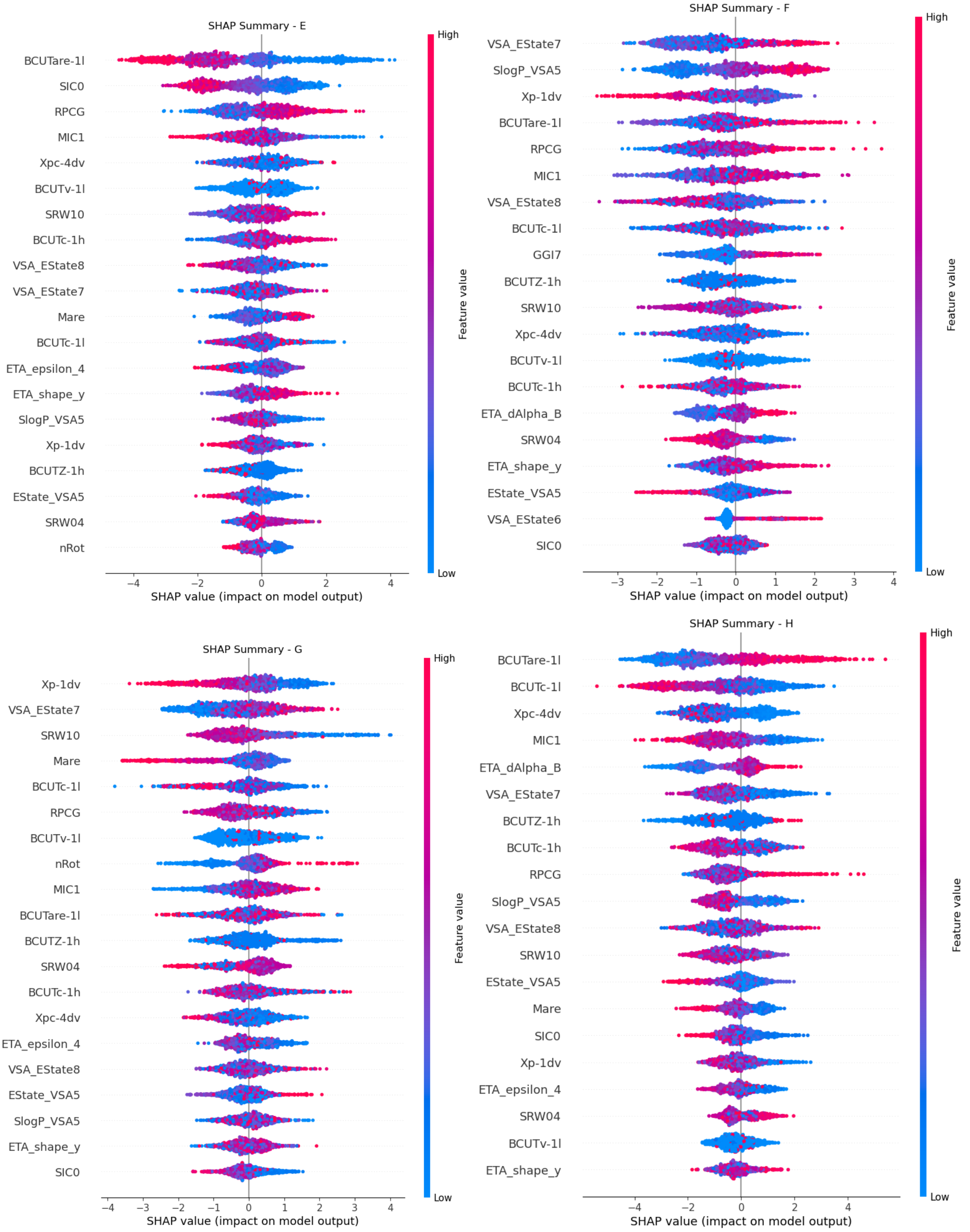

Figure S20-2: SHAP analysis of the XGBoost classifier on the DT for all 16 classes. See also manuscript for the available code on the GitHub repository.

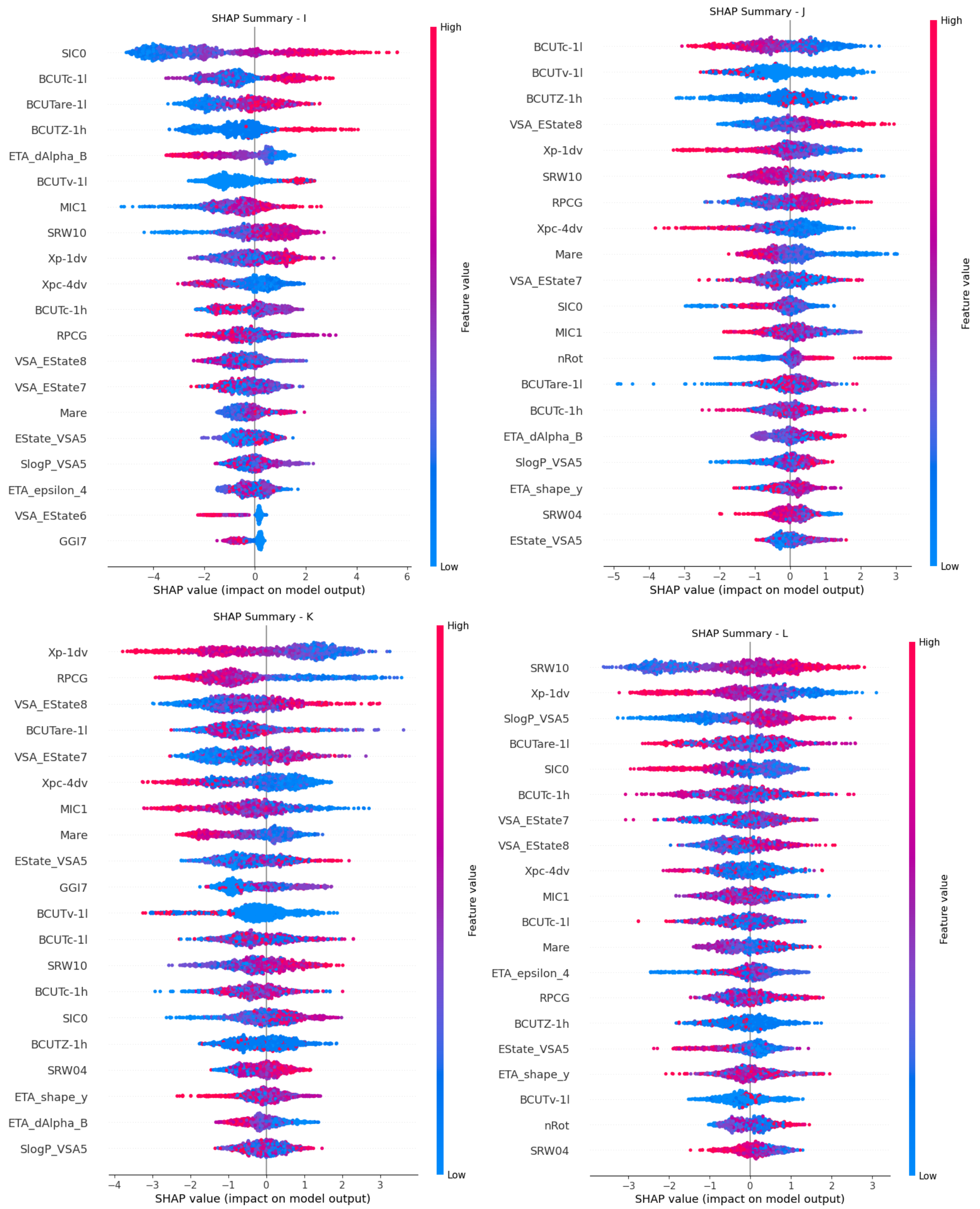

Figure S20-3: SHAP analysis of the XGBoost classifier on the DT for all 16 classes. See also manuscript for the available code on the GitHub repository.

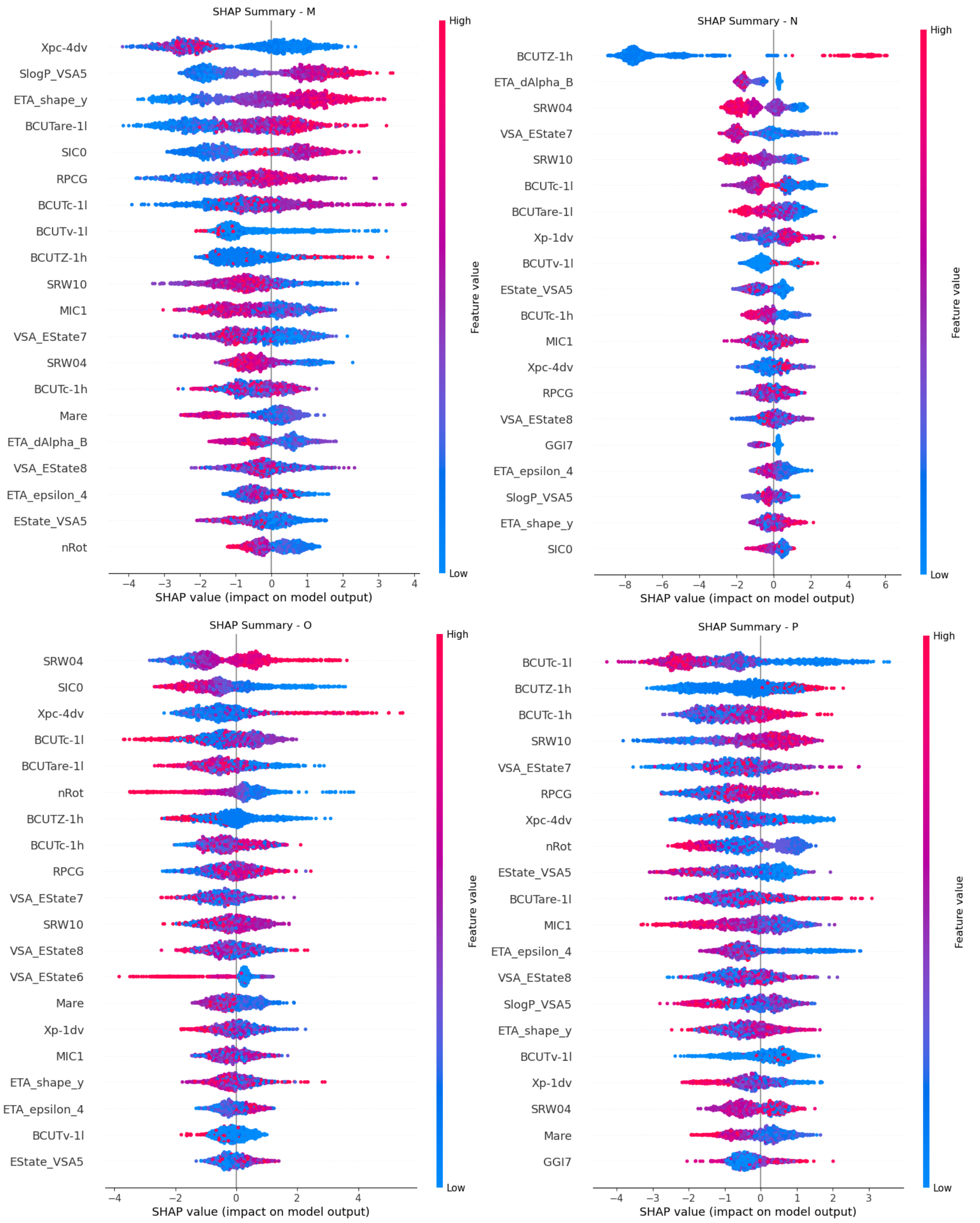

Figure S20-4: SHAP analysis of the XGBoost classifier on the DT for all 16 classes. See also manuscript for the available code on the GitHub repository.

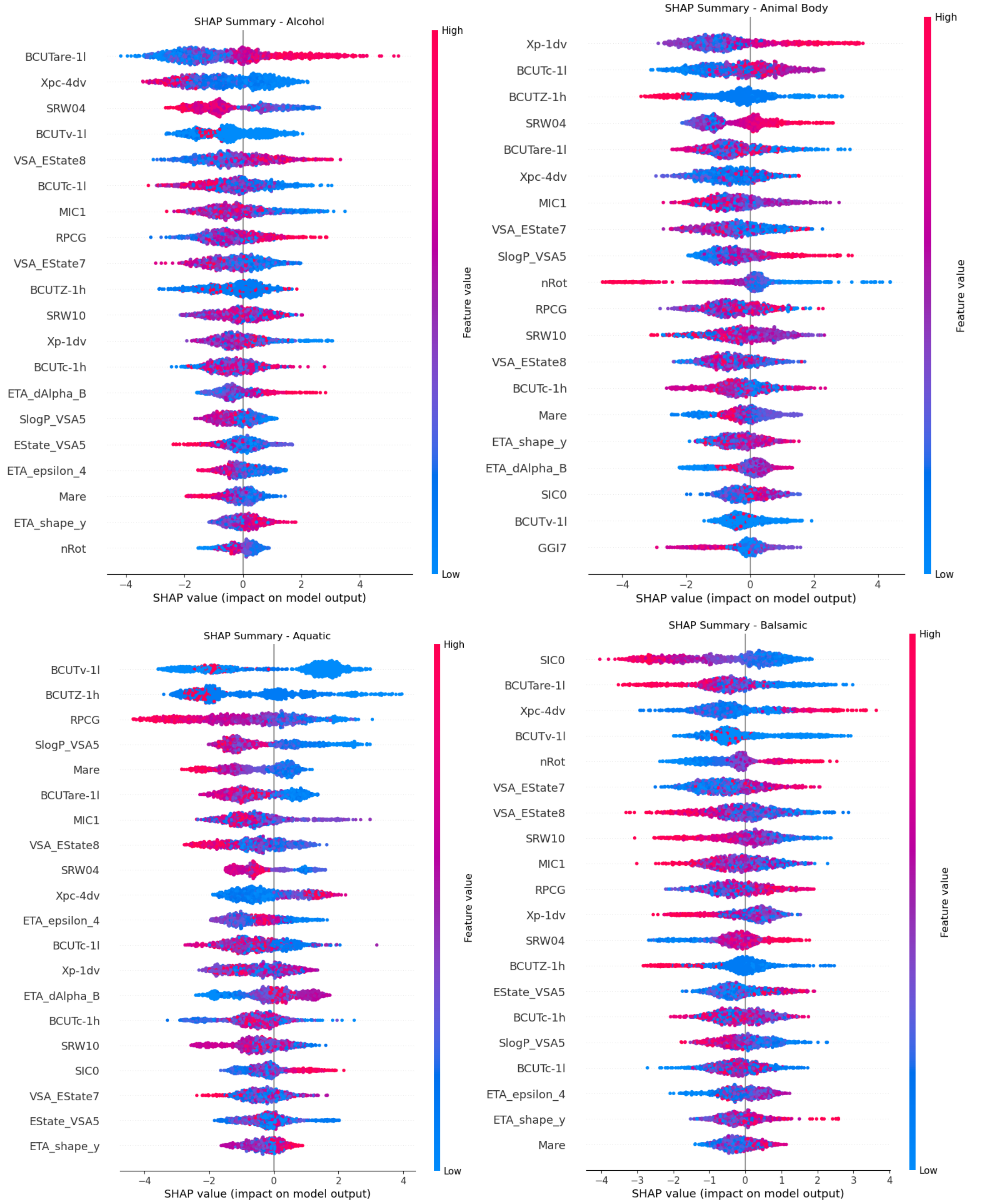

Figure S21-1. SHAP analysis of the XGBoost classifier on the expert taxonomy for all 16 classes. See also manuscript for the available code on the GitHub repository.

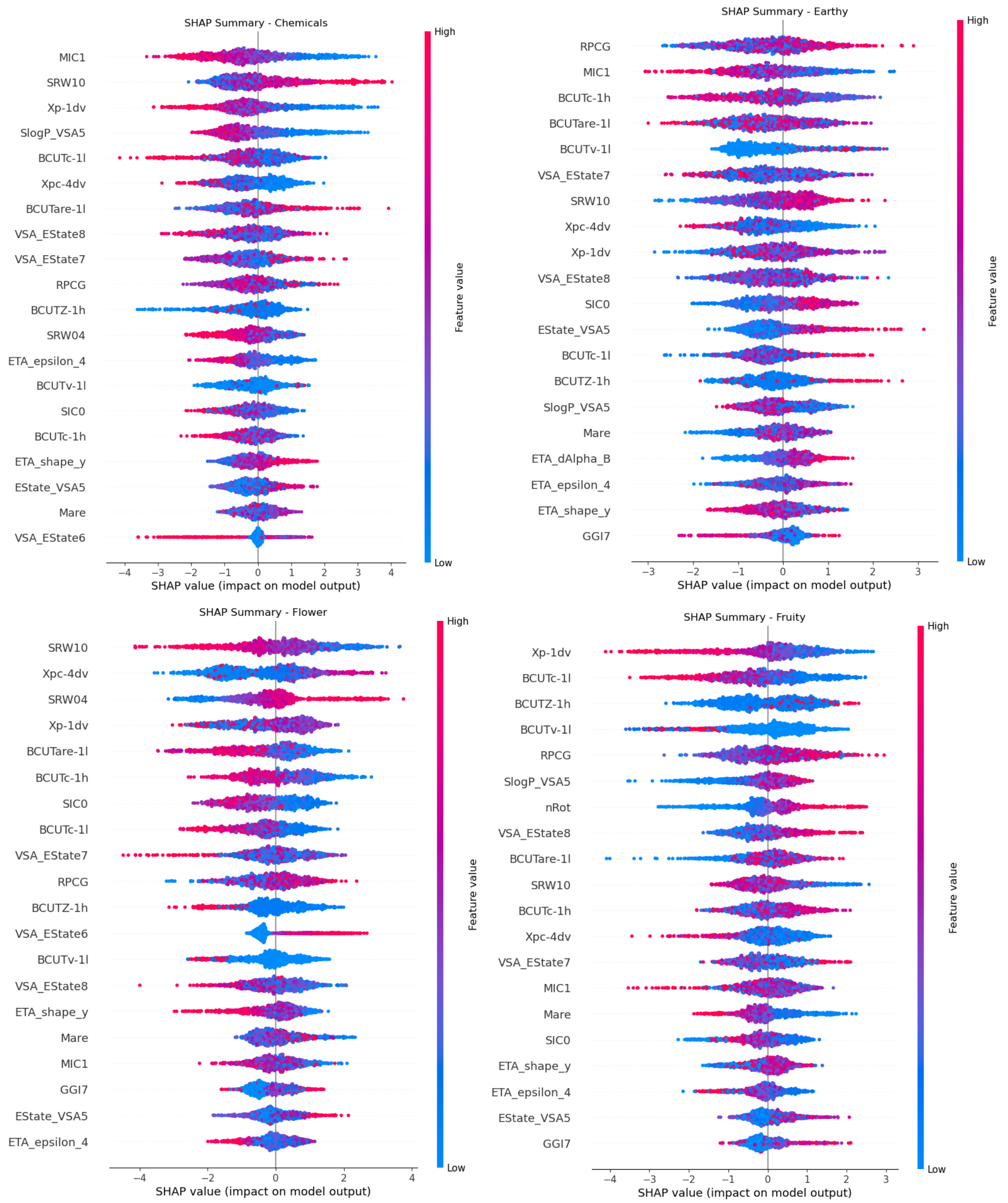

Figure S21-2. SHAP analysis of the XGBoost classifier on the expert taxonomy for all 16 classes. See also manuscript for the available code on the GitHub repository.

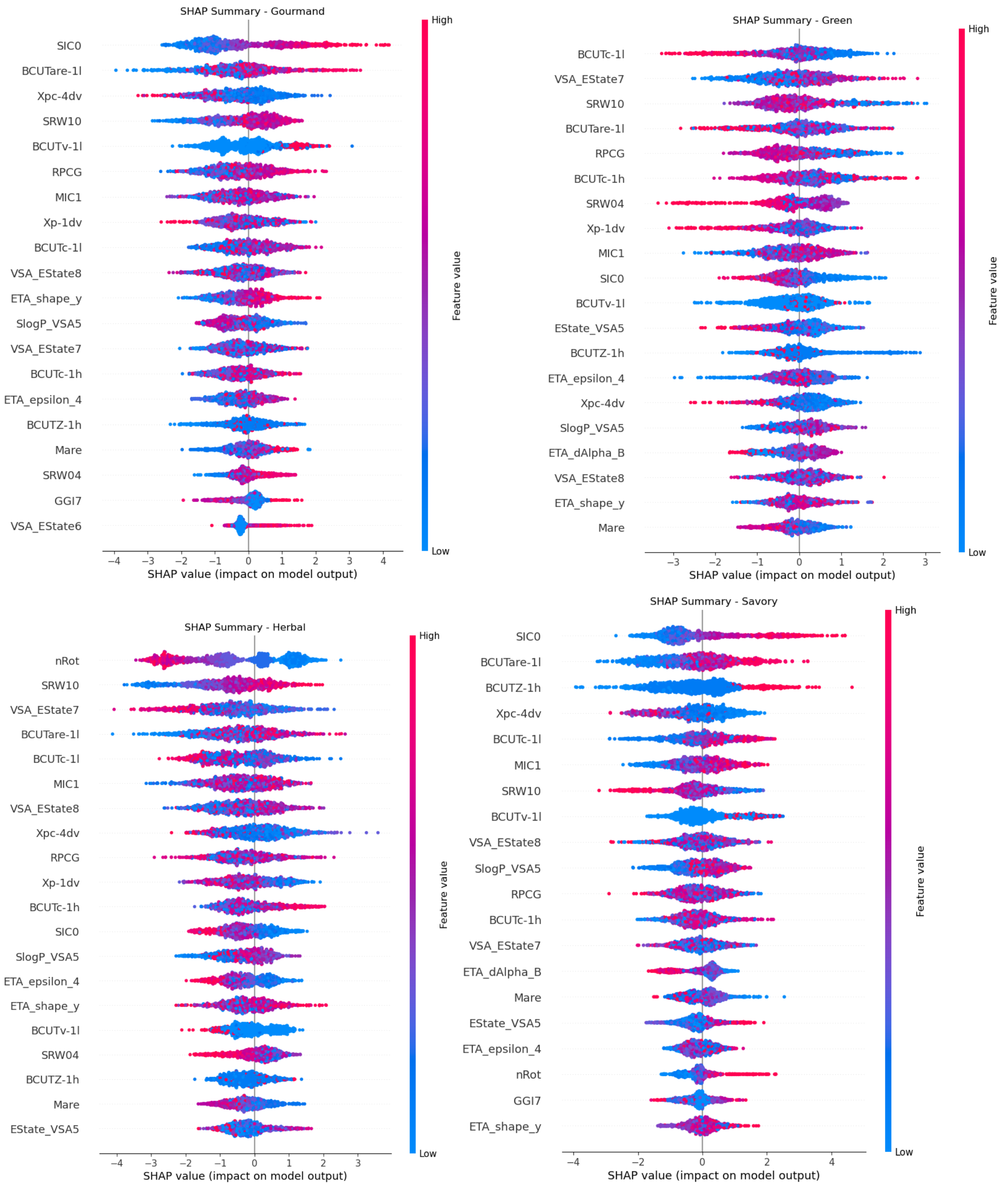

Figure S21-3. SHAP analysis of the XGBoost classifier on the expert taxonomy for all 16 classes. See also manuscript for the available code on the GitHub repository.

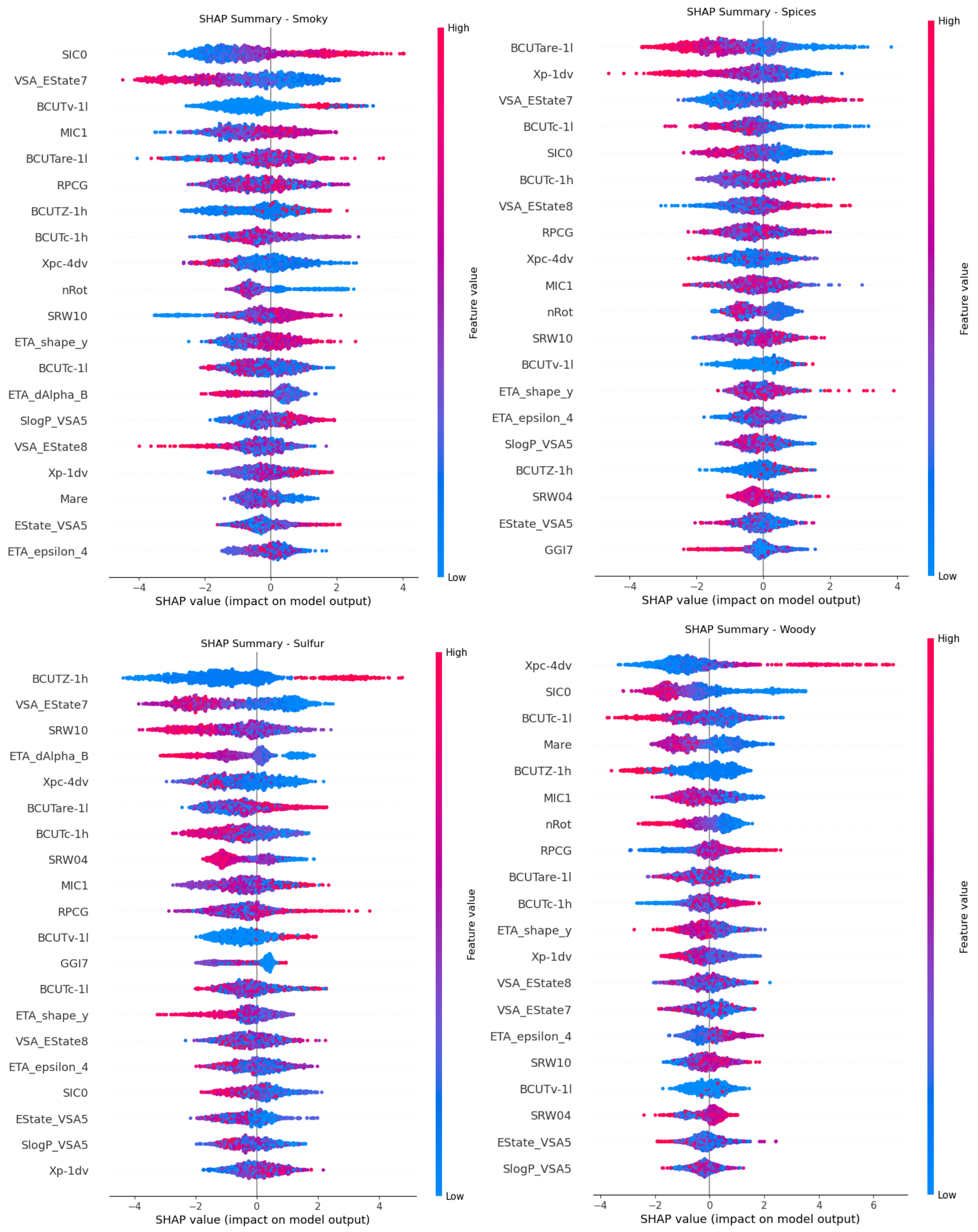

Figure S21-4. SHAP analysis of the XGBoost classifier on the expert taxonomy for all 16 classes. See also manuscript for the available code on the GitHub repository.

REFERENCES

[HEA] Head, T., Kumar, M., Nahrstaedt, H., Louppe, G., & Shcherbatyi, I. (2020). scikit-optimize/scikit-optimize (v0.8.1). Zenodo. https://doi.org/10.5281/zenodo.4014775

[LEE] Lee, B. K. *et al*. "A principal odor map unifies diverse tasks in olfactory perception", Science 381, 999–1006 (2023).

[LUN] Lundberg, Scott M., and Su-In Lee. "A Unified Approach to Interpreting Model Predictions", *Neural Information Processing Systems*, Curran Associates, Inc., 2017

[KWA] Kwak, S. G. & Kim, J. H. "Central limit theorem: the cornerstone of modern statistics", Korean J. Anesthesiol. 70, 144–156 (2017).
